# Novel Hypoxia-Associated Gene Signature Depicts Tumor Immune Microenvironment and Predicts Prognosis of Colon Cancer Patients

**DOI:** 10.3389/fgene.2022.901734

**Published:** 2022-06-06

**Authors:** Yixin Xu, Can Cao, Ziyan Zhu, Yibo Wang, Yulin Tan, Xuezhong Xu

**Affiliations:** ^1^ Department of General Surgery, Wujin Hospital Affiliated with Jiangsu University, Changzhou, China; ^2^ Department of General Surgery, The Wujin Clinical College of Xuzhou Medical University, Changzhou, China; ^3^ Department of General Surgery, Shanghai General Hospital of Nanjing Medical University, Shanghai, China; ^4^ Department of General Surgery, Shanghai General Hospital, Shanghai Jiao Tong University School of Medicine, Shanghai, China

**Keywords:** colon cancer, hypoxia, tumor immune microenvironment, prognostic model, overall survival

## Abstract

Hypoxia, a typical hallmark of numerous tumors, indicates poor infiltration of antitumor lymphocytes, as well as facilitates the development, progression, and drug resistance of malignant cells. Here, the present research was performed to identify novel hypoxia-related molecular markers and their correlation to the tumor immune microenvironment (TIME) in colon cancer. The expression of hypoxia-related gene signature was extracted from The Cancer Genome Atlas (TCGA) COAD cohort. Based on this signature, a risk score model was constructed using the Lasso regression model. Its discrimination ability and stability were validated in another independent cohort (GSE17536) from Gene Expression Omnibus (GEO) database. Moreover, molecular biology experiments (quantitative real-time PCR and multiple immunohistochemistry) were performed to validate the results of bioinformatics analyses. Three hub genes, including PPFIA4, SERPINE1, and STC2, were chosen to build the risk score model. All of these genes were increasingly expressed in the hypoxia subgroup (HS). Compared with the normoxia subgroup (NS), HS had worse pathological features (T, N, M, and stage) and overall survival (OS), more expression of immune checkpoint molecules, poorer infiltration of some pro-inflammation immune cells (CD4^+^ T cells and CD8^+^ T cells), and enriched infiltration of M0/M2 macrophages. After the risk model was proven to be valuable and stable, a nomogram was built based on this model and some clinicopathological factors. Moreover, it had been identified that three hub genes were all increasingly expressed in hypoxic conditions by quantitative real-time PCR (qPCR). The results of multiple immunohistochemistry (mIHC) also showed that higher expression of hub genes was associated with poorer infiltration of pro-inflammation immune cells (CD8^+^ T cells and M1 macrophages) and richer infiltration of anti-inflammation immune cells (Treg cells and M2 macrophages). In conclusion, the present study uncovered the relations among hypoxia, TIME, and clinicopathological features of colon cancer. It might provide new insight and a potential therapeutic target for immunotherapy.

## Introduction

Colon cancer (CC) is one of the most common malignancies worldwide and responsible for more than 0.5 million deaths in 2020 (Sung et al., 2021). Compared with 2018, the numbers of new cases and deaths of CC have rapidly increased (Bray et al., 2018; Sung et al., 2021). Despite the advance in medical technology, patients with CC still have a relatively high mortality rate, 13.1% in the transitioning and 4.7% in the transitioned countries (Sung et al., 2021). Owing to the heavily medical and financial burden caused by CC, it is urgent to develop novel methods to improve the diagnostic and therapeutic efficiency for these patients.

Recently, immunotherapy has attached much attention from the public for its promising therapeutic efficiency. It is well established that the killing effect induced by immunotherapy relies on some immune cells that are recognized as tumor suppressors (Hiam-Galvez et al., 2021). While in the complex tumor microenvironment (TME), besides these tumor suppressors, there are many other components that promote the development and progression of cancer (Anderson and Simon, 2020). These promoters and the tumor itself deprive of the oxygen and nutrient and subsequently produce a hypoxic and acidic TME, which significantly restrain the function of those antitumor immune cells (Kaymak et al., 2021). Previous studies have well established that hypoxia is a hallmark of tumor growth, survival, and metastasis of CC and confers to resistance to immunotherapy (Hsu et al., 2020; Singhal et al., 2021). Therefore, the establishment of a hypoxia-related gene signature may help to comprehend the immunogenomic profile of CC and provide a useful prognostic tool for CC patients.

Here, based on The Cancer Genome Atlas (TCGA) and the Gene Expression Omnibus (GEO) databases, we developed a hypoxia-related gene signature to depict the tumor immune microenvironment (TIME) and predict the overall survival (OS) of CC patients. Moreover, we also did quantitative real-time PCR (qPCR) and multiple immunohistochemistry (mIHC) to verify the results of bioinformatics analyses.

## Materials and Methods

### Training and Validation Cohort

The FPKM RNA-seq data (398 tumors and 39 normal tissue samples) and related clinical information of 385 CC patients were obtained from the TCGA database (https://portal.gdc.cancer.gov/) using the GDC API tools on 7 July 2021. Then, 282 patients with complete overall survival (OS) data were included in the training set. Meanwhile, the GSE17536 cohort was applied as the independent validation set. It included 177 colon tumor tissue samples and was obtained from the GEO database (https://www.ncbi.nlm.nih.gov/geo/query/acc.cgi?acc=gse17536). The FPKM RNA-seq data from TCGA were transformed to log_2_(x+1). Then, they were normalized to eliminate the batch effect using *limma* (version 3.48.1) and *sva* (version 3.40.0) packages.

### Gene Signature List

200 hypoxia-related genes were accessed from the HALLMARK_HYPOXIA gene set of Gene set Enrichment Analysis (GSEA) database (http://www.gsea-msigdb.org/gsea/msigdb/cards/HALLMARK_HYPOXIA.html). The complete gene list was contained in Supplementary Table S1.

The immune-related genes were obtained from the Tracking Tumor Immunophenotype database (http://biocc.hrbmu.edu.cn/TIP/index.jsp) (Xu et al., 2018). This gene list contained negative regulatory, positive regulatory, T cell, CD8^+^ T cell, CD4^+^ T cell, dendritic cell, eosinophil, macrophage, monocyte, neutrophil, nature kill (NK) cell, Th1 cell, Th17 cell, Th 2 cell, Th22 cell, and Treg cell-correlated genes.

### Procedure of Developing Risk Score Model

First, the fold change (FC) of the 200 hypoxia-related genes between tumor and normal tissue samples was calculated using the *limma* package. Genes with log_2_|FC|>1 & adjusted *p*-value < 0.05 were identified as the differentially expressed genes (DEGs). Meanwhile, the statistically prognostic genes were identified using univariate Cox regression analysis. Then, the DEGs (Supplementary Table S2) and prognostic genes (Supplementary Table S3) were intersected to identify hub genes.

Based on the least absolute shrinkage and selection operator (LASSO) regression analysis, the formula of the risk score model was built as follows:

$$\text{Risk score model}=\underset{i}{\sum }\beta_{i}\;*\;hub\;gene_{i}$$

The i index represents a significantly prognostic gene of the Lasso regression analysis and $\beta_{i}$ stands for the beta coefficients of these genes.

### Differences in RNA Expression and Clinical Characteristics Between Subgroups

To compare the differences between subgroups in RNA expression/gene function/clinical characteristics, we used *Rtsne* (version 0.15) and *pheatmap* (version 1.0.12) packages/GSEA analysis/*stats* (version 4.1.0) package.

### Evaluation of Tumor Immune Microenvironment and Drug Response

To investigate the association between tumor immune microenvironment (TIME) and the risk score model, we used CIBERSORT and microenvironment cell populations-counter (MCP-counter) to estimate the infiltration of different immune cells (Newman et al., 2019; Becht et al., 2016). While for single-gene analysis, we used the TIMER webserver to evaluate the relation between six different types of immune cells and the target gene (https://cistrome.shinyapps.io/timer/) (Li et al., 2016; Li et al., 2017).

To evaluate the response of immune checkpoint blockade (ICB), we used the website tool ImmuCellAI (http://bioinfo.life.hust.edu.cn/ImmuCellAI#!/), which was based on ssGSEA analysis (Miao et al., 2020; Miao et al., 2021). But the *pRRophetic* package (version 0.5) was applied to compare the differences in drug response for cytotoxic and targeted medicine (Geeleher et al., 2014).

### Model Visualization, Validation, and Comparison

To visualize the risk score model, we used the *rms* package (version 6.1-0) to create a nomogram that could predict the 1-, 3-, and 5-year OS of CC patients. It contained clinical factors (age, pathological M, and stage) and the risk score.

The discrimination ability of the risk score model was assessed using operating characteristic curve (ROC) analysis. Based on a series of different binary classification methods (critical or cutoff value), it could calculate the true positive (TP) and false positive (FP). The curve was drawn with TP or sensitivity as the ordinate, and with FP or 1-specificity as the abscissa. The area under the curve (AUC) was used for quantitative analysis in ROC analysis. Generally (Sung et al., 2021) AUC between 0.5 and 0.7 would be considered lower accuracy (Bray et al., 2018); AUC between 0.7 and 0.9 would be considered to be valuable (Hiam-Galvez et al., 2021); AUC above 0.9 would be considered high accuracy; however, AUC = 0.5 meant that the model had no diagnostic value. In this study, ROC analysis was performed using the *timeROC* package (version 0.4).

The calibration plot was used to assess the 1-, 3-, and 5-year OS, based on the Cox proportional hazard model. Meanwhile, the Kaplan-Meier (K-M) survival analysis was performed according to different subgroups (NS vs. HS) in both training and validation sets. The calibration and K-M analysis were performed using the *rms* and *survival* (version 3.2-7) packages.

In this study, Decisive Curve Analysis (DCA) was used to estimate prediction ability among different models. The abscissa of the DCA plot was threshold probability, and the ordinate was the net benefit (NB) after therapeutic advantages minus the disadvantage. In general, the farther the curve was from the extreme curves the better its prediction power represented. The DCA analysis was performed using the *ggDCA* (version 1.2).

### Cell Culture and Culture Conditions

The human colon epithelial cell line (FHC) and colon cancer cell lines (HCT-8, RKO, SW480, and SW620) used *in vitro* experiment was purchased from the Cell Bank of the Chinese Academy of Science and authenticated by the supplier. These cell lines were grown in DMEM (Gibco) supplemented with 10% fetal calf serum (FCS). We have identified the source of cell lines by STR profiling. Meanwhile, the cells were routinely tested for mycoplasma contamination (MycoAlert PLUS Mycoplasma Detection Kit, Lonza).

In normoxic conditions, cell lines were maintained at 37°C in the humidified incubator with 5% CO_2_ (Thermo Scientific). Cell lines would be plated at the desired density (60%–70%) 24 h before the placement into a hypoxia incubator (BioSpherix). The condition of hypoxia treatment was set to 1% O_2_ and 5% CO_2_ for 24 h.

### RNA Extraction and qRT-PCR

RNA was isolated using TRI reagent solution (Sigma) followed by the on-column RNeasy mini kit and DNase treatment (Qiagen, Germany). cDNA synthesis was performed using the Transcription First Strand cDNA Synthesis Kit (Roche). qRT-PCR was performed using ABI 7900T PCR System (Applied Biosystems). Gene expression using SYBR Magic was normalized to the expression of β-actin. The primers used in the present study were supplied in Supplementary Table S4.

### Multiple Immunohistochemistry Staining

Four-micron slices, cut from the paraffin block of tissues, were mounted onto charged slides and baked at 60°C for 1 h as the first step. Then, they were dewaxed with xylene for 10 min and stained with 100%, 90%, and 70% ethanol for 10 min per concentration. After being washed with deionized water for 2 min, these slides would be soaked in neutral buffered formalin for 30 min. Next, Opal manual kit (PerkinElmer) was used to stain the slides according to the manufacturer’s instructions. After nonspecific antigen sites were blocked, slides were incubated with antigen-specific primary antibody overnight at 4°C; secondary antibody incubation was performed for 1 h at room temperature. Then, dyes contained in the kit (Opal TSA) would be applied for immunofluorescence staining. We found slide stained with 3 markers plus 4 colors was the optimal choice. AR9 buffer would be used for antigen retrieval after three steps (incubation of primary antibody, secondary antibody, and dye staining) were finished. Finally, the slides were incubated with DAPI for nuclear DNA staining.

All of the primary antibodies used in mIHC are listed in Supplementary Table S5.

### Statistical Analysis

Continuous and categorical (frequencies and percentages) variables were analyzed using independent t, chi-square, or 2-tailed Fisher exact tests, respectively. Meanwhile, ranked data were analyzed using the Mann-Whitney U test. The discrimination of the prediction model was assessed using ROC analysis. The OS was defined as the period from the date of surgery to the date of death due to any cause. OS between different groups was measured using the Log-rank method of K-M analysis. Cox regression analysis was used to assess time-event-dependent OS status of CC patients. The correlations of RNA expression among different hub genes were measured using spearman analysis. A *p*-value less than 0.05 was considered statistically significant. All statistical analyses were carried out using R (version 4.0.3; https://www.r-project.org/) and R studio (version 1.3.1093; https://www.rstudio.com/) software.

## Results

### Baseline Characteristics of Training and Validation Sets

All detailed information on baseline characteristics of both training and validation sets are listed in Table 1.

**TABLE 1:** Different characteristics between low- and high-risk groups in the training (TCGA) and validation (GEO) sets.

Characteristics	TCGA					GEO				
	Total(271)	High risk(135)	Low risk(136)	t/X^2^/Z	*P*	Total(177)	High risk(88)	Low risk(89)	t/X^2^/Z	*p*
BMI	—	28.23 ± 5.96	31.95 ± 26.47	1.361	0.175	—	—	—	—	—
Position	—	—	—	2.812	0.422	—	—	—	—	—
Right colon	134	65	69	—	—	—	—	—	—	—
Transverse colon	22	8	14	—	—	—	—	—	—	—
Left colon	17	10	7	—	—	—	—	—	—	—
Sigmoid colon	93	50	43	—	—	—	—	—	—	—
N/A	5	2	3	—	—	—	—	—	—	—
Lymph node	—	2.45 ± 5.27	0.98 ± 1.98	2.975	0.003	—	—	—	—	—
Tumor history	—	—	—	0.361	0.548	—	—	—	—	—
Yes	90	48	42	—	—	—	—	—	—	—
No	158	78	80	—	—	—	—	—	—	—
N/A	23	9	14	—	—	—	—	—	—	—
CEA	—	41.67 ± 161.07	38.00 ± 268.81	0.114	0.909	—	—	—	—	—
Venous invasion	—	—	—	—	—	—	—	—	—	—
Yes	62	43	19	10.203	0.001	—	—	—	—	—
No	183	84	99	—	—	—	—	—	—	—
N/A	26	8	18	—	—	—	—	—	—	—
Lymphatic invasion	—	—	—	3.371	0.066	—	—	—	—	—
Yes	93	55	38	—	—	—	—	—	—	—
No	157	74	83	—	—	—	—	—	—	—
N/A	21	6	15	—	—	—	—	—	—	—
Perineural invasion	—	—	—	—	—	—	—	—	—	—
Yes	36	25	11	4.710	0.030	—	—	—	—	—
No	107	52	55	—	—	—	—	—	—	—
N/A	128	58	70	—	—	—	—	—	—	—
History of colon polyps	—	—	—	0.495	0.482	—	—	—	—	—
Yes	72	34	38	—	—	—	—	—	—	—
No	157	82	75	—	—	—	—	—	—	—
N/A	42	19	23	—	—	—	—	—	—	—
Colon polyps	—	—	—	1.900	0.168	—	—	—	—	—
Yes	64	29	35	—	—	—	—	—	—	—
No	116	65	51	—	—	—	—	—	—	—
N/A	91	41	50	—	—	—	—	—	—	—
dMMR	—	—	—	6.322	0.012	—	—	—	—	—
Yes	48	17	31	—	—	—	—	—	—	—
No	153	86	67	—	—	—	—	—	—	—
N/A	70	32	38	—	—	—	—	—	—	—
Gender	—	—	—	0.295	0.587	—	—	—	0.048	0.826
Female	124	64	60	—	—	81	41	40	—	—
Male	147	71	76	—	—	96	47	49	—	—
Age	—	67 ± 12.76	64.85 ± 12.59	1.418	0.157	—	63.18 ± 14.17	67.75 ± 11.55	2.354	0.020
*Stage	—	—	—	18.733	0.000	—	—	—	14.901	0.002
Ⅰ	47	12	35	—	—	24	6	18	—	—
Ⅱ	106	50	56	—	—	57	23	34	—	—
Ⅲ	79	47	32	—	—	55	37	18	—	—
Ⅳ	39	26	13	—	—	41	22	19	—	—
*T	—	—	—	14.236	0.003	—	—	—	—	—
T1	8	1	7	—	—	—	—	—	—	—
T2	45	14	31	—	—	—	—	—	—	—
T3	190	102	88	—	—	—	—	—	—	—
T4	28	18	10	—	—	—	—	—	—	—
*N	—	—	—	16.819	0.000	—	—	—	—	—
N0	160	65	95	—	—	—	—	—	—	—
N1	68	38	30	—	—	—	—	—	—	—
N2	43	32	11	—	—	—	—	—	—	—
M	—	—	—	—	—	—	—	—	—	—
M0	232	109	123	5.175	0.023	—	—	—	—	—
M1	39	26	13	—	—	—	—	—	—	—
Race	—	—	—	0.681	0.712	—	—	—	2.165	0.539
Asian	8	3	5	—	—	17	7	10	—	—
Black	47	24	23	—	—	9	4	5	—	—
White	170	89	81	—	—	151	77	74	—	—
Others	46	19	27	—	—	—	—	—	—	—
*Grade	—	—	—	—	—	—	—	—	7.307	0.026
1	—	—	—	—	—	21	7	14	—	—
2	—	—	—	—	—	119	56	63	—	—
3	—	—	—	—	—	37	25	12	—	—

Note: *These variables were ranked data, so they were compared between different subgroups using the Mann-Whitney rank sum test.

In the training set, 271 CC patients had complete clinical and pathological data and the remaining 11 patients only had follow-up information. Among the 271 patients, 124 patients were female (43.97%) and 147 were male (52.13%). The average age was 65.06 ± 12.70 years. Meanwhile, 224 patients (79.43%) had advanced disease (Stages II–IV), among them 39 patients (13.83%) with distant metastasis.

Also in the validation set, data of average age, the constituent ratio of gender, and pathological stage were provided. The average age and constituent ratio of pathological were comparable between the two sets. The information on pathological grade was only available in the validation set. There were 16 patients with grade 1 (9.04%), 134 with grade 2 (75.71%), and 27 with grade 3 (15.25%) disease.

### Searching Procedure of Hub Hypoxia-Related Genes and Development of Risk Score Model

**FIGURE 1:**
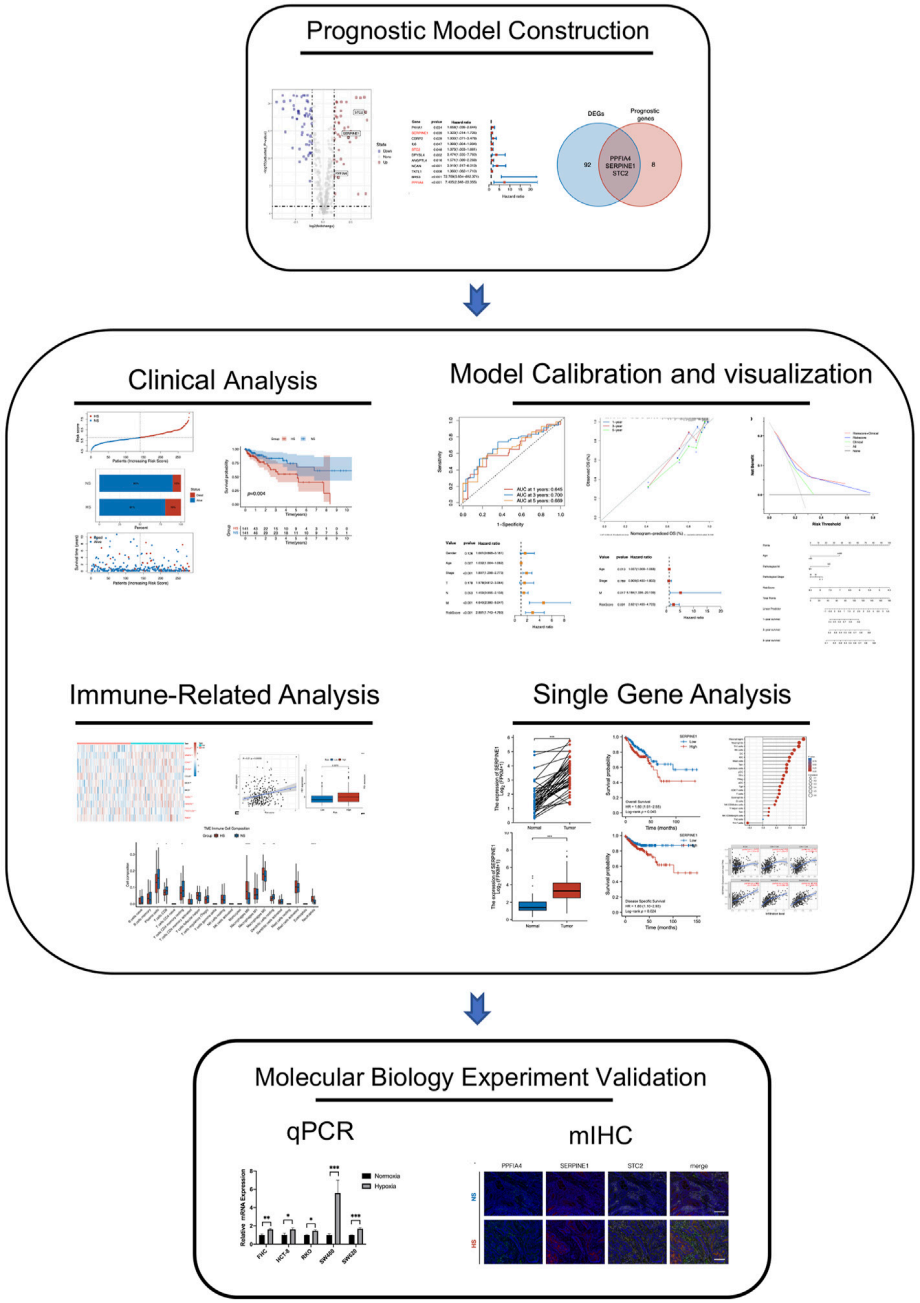
The complete pipeline of the present study. It contained three major parts, including prognostic model construction, analyses between different subgroups (clinical, immune-related, calibration, bio-functional, and single-hub gene analyses), and multiple immunohistochemistry validation.

The complete pipeline of this study is shown in Figure 1.

We defined hub hypoxia-related genes as those that were differentially expressed between tumor and normal tissue samples and were statistically associated with the prognosis of CC patients.

**FIGURE 2:**
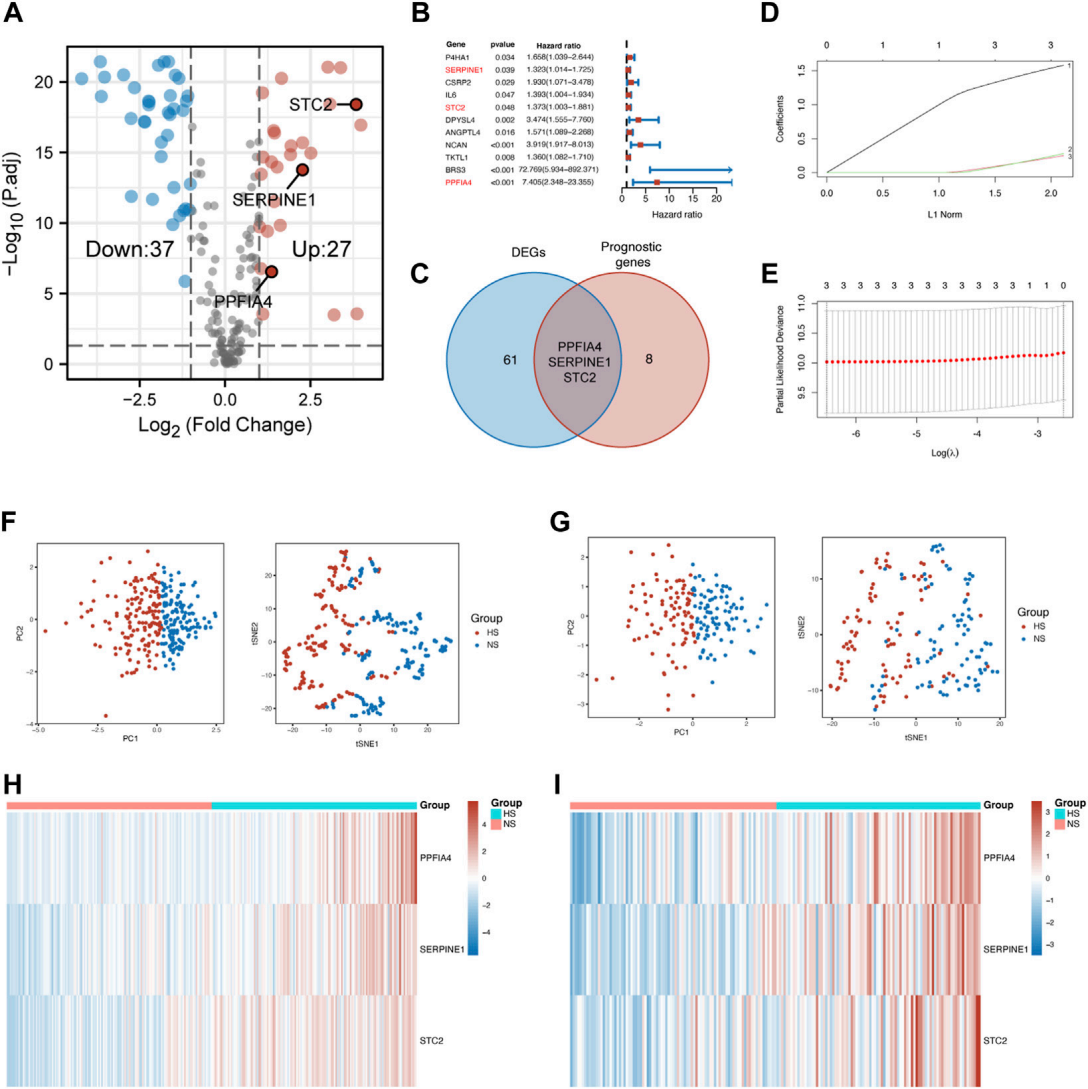
Construction of the prognostic model. **(A)** The volcano plot showed hypoxia-related DEGs extracted from the TCGA-COAD cohort. **(B)** The forest plot displayed the hypoxia-related prognostic genes extracted from the TCGA-COAD cohort. Three hub genes were marked in red font. **(C)** The Venn diagram showed that the intersection of DEGs and prognostic genes were three hub genes, including PPFIA4, SERPINE1, and STC2. **(D)** 20-Time cross-validation for tuning parameter selection in the LASSO Cox model. The plot of LASSO coefficients **(E)** showed the best choice of the number of these genes was 3. The PCA and t-SNE scatter plots confirmed that the risk score model could precisely classify patients into two different groups in the training **(F)** and validation **(G)** sets. The heatmap visualized different expression patterns of hub genes in NS and HS in the training **(H)** and validation **(I)** sets. DEGs: differentially expressed genes.

According to these criteria, we first extracted the expression of 200 hypoxia-related genes from the TCGA-COAD cohort. Second, 64 DEGs (Figure 2A) and 11 prognostic genes (Figure 2B) were identified. Then, three hub genes (PPFIA4, SERPINE1, and STC2) were established as the intersection of DEGs and prognostic genes (Figure 2C).

After the confirmation of hub genes, Lasso regression analysis was used to construct the risk score model (Figures 2D,E). The formula was built as follows:

Risk score = 1.582 × PPFIA4 + 0.249×SERPINE1 + 0.279 × STC2

After the risk score of each patient was calculated, these patients were divided into two subgroups, according to the median of the risk score. The normoxia subgroup (NS) represented the one having a lower expression level of hub genes, while the hypoxia subgroup (HS) was defined as the one with a higher expression level of hub genes.

To evaluate the distinguishing ability of the risk score, principal component analysis (PCA), t-SNE (t-distributed stochastic neighbor embedding), and heatmap were used. The results of the PCA and t-SNE methods suggested that two subgroups could be separated clearly and stably according to the risk score in the training (Figure 2F) and validation (Figure 2G) sets. Figures 2H,I shows that the expression of all three hub genes was also higher in the HS compared with that in the NS in both sets.

After the grouping method was proven to be acceptable, we then investigated the differences in clinicopathological features, survival, and immune status between the two subgroups.

### Different Clinicopathological and Prognostic Characteristics

First, we compared clinicopathological characteristics between two subgroups in both training and validation sets. In the training set, we found that there were more patients with metastatic lymph nodes, venous invasion, lymphatic invasion, perineural invasion, and proficient mismatch repair (pMMR) in the HS. Meanwhile, these patients had the worse pathological stage, T (invasion depth), N (lymph node metastasis), and M (distant metastasis). In the validation set, patients in the HS had poorer pathological stage and grade, and younger age (Table 1).

**FIGURE 3:**
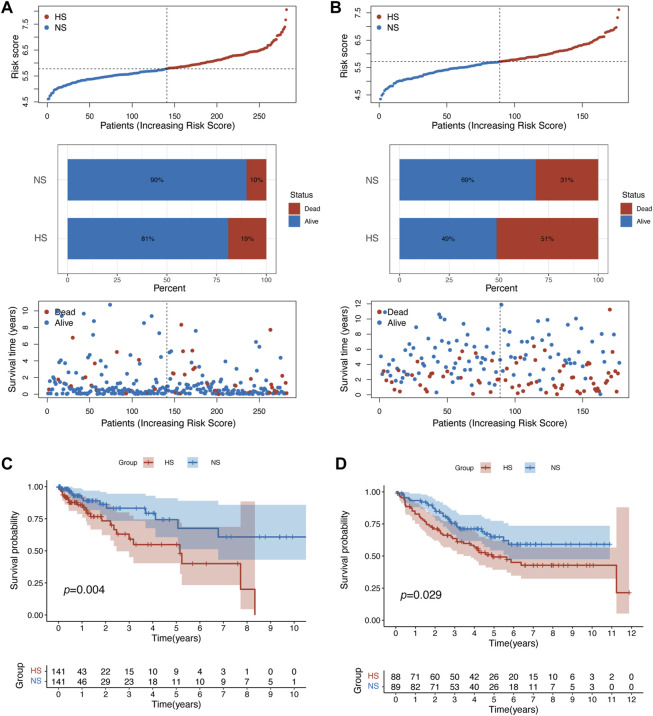
Patients in different subgroups showed statistically different prognoses. **(A)** The patient distribution, risk score, and status plots showed that patients in the HS in training set related to poorer prognosis. **(B)** Similar results were identified in the validation set. The survival plot of K-M analysis confirmed that patients in HS had statistically poorer overall survival in the training **(C)** and validation **(D)** sets.

Second, compared with NS, HS had a higher mortality rate (19% vs. 10% in the training set; 51% vs. 31% in the validation set) (Figures 3A,B). The results of the K-M analysis suggested that patients in HS had significantly poorer OS in both training (*p* = 0.004) and validation (*p* = 0.029) sets (Figures 3C,D).

### The Validation of Discrimination Ability and Stability of the Risk Score Model

**FIGURE 4:**
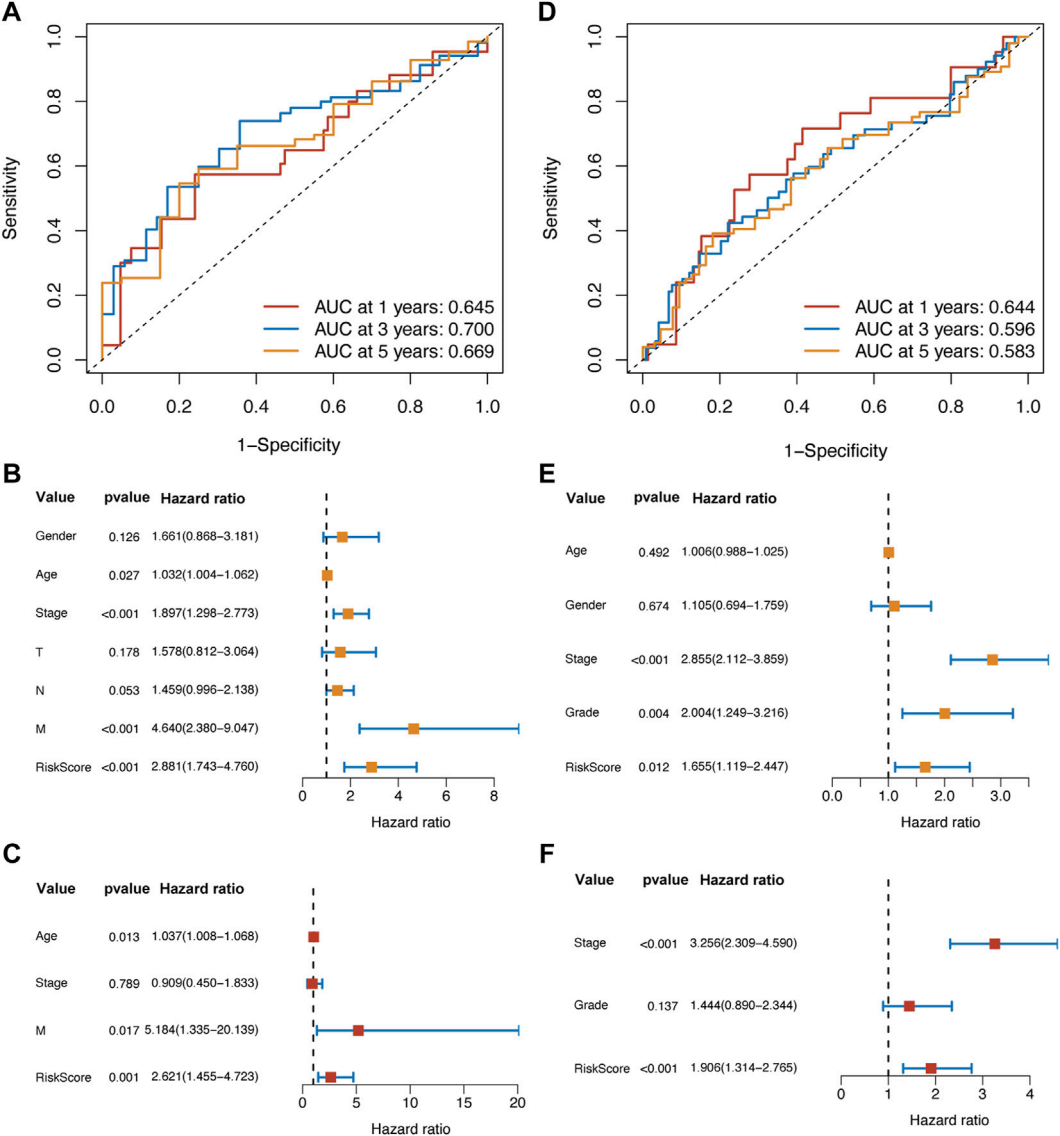
The ROC analysis suggested that the prognostic ability of the risk score model was valuable and stable in different survival durations in the training set **(A)** and validation set **(D)**. In the training set, the univariate **(B)** and multivariate Cox **(C)** analysis showed that the risk score was an independent prognostic factor. Meanwhile, the results of Cox analysis in the validation set **(E,F)** were following those in the training set.

In the training set, the results of the ROC analysis showed that the AUCs of our risk score model were larger than 0.6 (1-year: 0.645; 3-year: 0.700; 5-year: 0.669) (Figure 4A). Then, we used Cox regression analysis to determine whether the risk score was an independent prognostic factor. The univariate Cox analysis suggested that age, pathological stage, distant metastasis, and the risk score were associated with the prognosis (Figure 4B). The multivariate Cox analysis identified that age, distant metastasis, and the risk score were the independent prognostic factors (Figure 4C).

In the validation set, the AUCs of 1, 3, and 5 years were all above 0.5 (1-year: 0.644; 3-year: 0.596; 5-year: 0.583) (Figure 4D). Through univariate and multivariate Cox analysis, we found that the pathological stage and the risk score were the independent prognostic factors (Figures 4E,F).

Taken together, the results confirmed that the risk score model based on the hub hypoxia-related genes had valuable discrimination ability and stability. It could predict the prognosis of patients with colon cancer accurately in the different study populations.

### Visualization and Calibration of the Risk Score Model

We decide to use the clinicopathological features (age, M, and pathological stage) in the TCGA-COAD cohort, along with risk score, to build a nomogram for clinical application.

**FIGURE 5:**
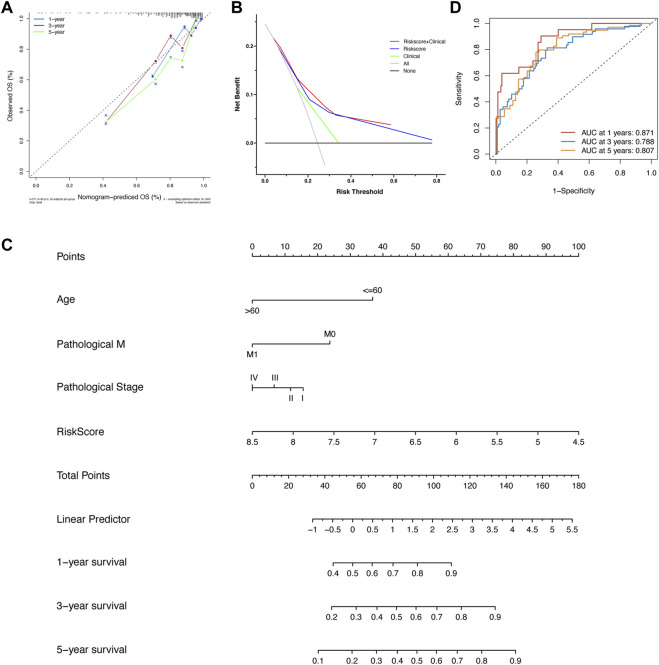
**(A)** The calibration analysis suggested that the prognostic performance of the risk score model was stable. **(B)** The DCA analysis showed that the prognostic ability of the risk score model plus clinical factors was best, followed by the risk score model alone and clinical factors alone. **(C)** The nomogram was built based on the risk score model and several clinical variates. **(D)** The ROC analysis of nomogram in the training set.

First, in Figure 5A, the result of calibration analysis showed that the blue, red, and green lines, which represented the performance of 1-, 3-, and 5-year prognostic prediction, were just close to the diagonal. It meant that the risk score was a stable prognostic model. Next, we compared the prognostic ability of three models, including the risk score, clinical characteristics (age, M, and pathological stage), and risk score plus clinical characteristics using Decisive Curve Analysis (DCA). The results showed that the performance of the multiple-factor model (risk score plus clinical characteristics) was slightly better than that of the single-factor model (the risk score model alone); however, both of their performance was significantly better than that of the sole clinical characteristics model (Figure 5B).

Since the diagnostic ability of the risk score model was validated to be valuable and stable, we then visualized it using a “nomogram” (Figure 5C). Patients with colon cancer could predict their 1-, 3-, and 5-year OS according to the information of age, pathological stage, status of distant metastasis, and our risk score model. It also could help doctors to predict the prognosis of patients with colon cancer accurately and easily. After the construction of the nomogram, we also evaluated the prediction power of the nomogram; the AUCs of 1-, 3-, and 5-year OS were 0.871, 0.788, and 0.807, respectively (Figure 5D).

Meanwhile, we also did calibration analysis, DCA, and ROC analysis, and built a nomogram in the validation set. The results showed that the risk model had stable prediction performance and the combined usage of the risk model with clinicopathological features had better prediction power than that of mono-marker (Supplementary Figure S1). The AUCs of 1-, 3-, and 5-year OS of nomogram in the validation set were 0.768, 0.724, and 0.676, respectively (Supplementary Figure S1D).

### Analyses of Differentially Expressed Genes and Functional Enrichment

**FIGURE 6:**
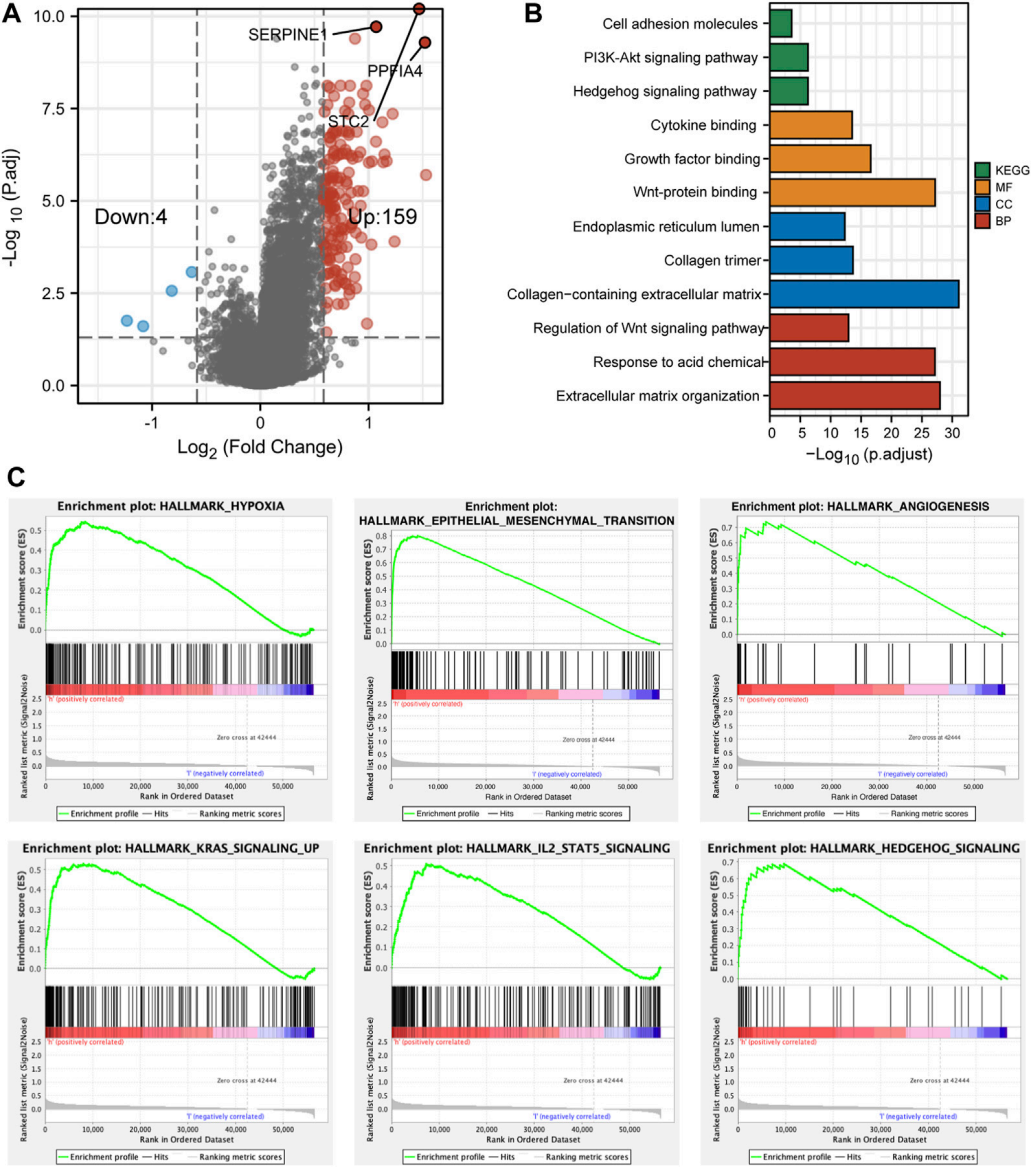
The differentially expressed genes and functional enrichment. **(A)** The volcano plot showed that 159 genes were highly expressed and 4 genes were lowly expressed in HS. **(B)** The bar plot revealed different functional enrichment involved in HS, according to the DEGs. **(C)** The results of GSEA analyses showed that hypoxia, EMT, angiogenesis, KRAS, IL2, and hedgehog pathways were enriched in HS.

After the confirmation of the correlation between risk score and the prognosis of CC patients, we then investigated different bio-functions between NS and HS. First, we used the *limma* package to identify DEGs between NS and HS. There were 72 DEGs (log_2_|FC|>1 & adjusted *p* < 0.05) between different subgroups. Because bio-functional analysis should be built on enough DEGs, we reset the standard of DEGs to |FC|>1.5. As a result, there were 163 DEGs (4 down and 159 up in the HS) (Figure 6A).

Through GO and KEGG analyses, we found that the bio-functions of upregulated genes in the HS were enriched in proliferation, differentiation, and tumorigenesis-related signaling pathways, including PI3K-Akt, Hedgehog, and Wnt signaling pathways. Meanwhile, these DEGs were also involved in Growth factor and Cytokine binding and extracellular matrix reorganization and reconstruction (Figure 6B). Moreover, GSEA analysis revealed that compared with NS, HS was enriched in hypoxia, epithelial–mesenchymal transition (EMT), angiogenesis, and KRAS, IL2/STAT5, and Hedgehog signaling pathways (Figure 6C).

Taken together, HS had enriched bio-functions in tumorigenesis, proliferation, and differentiation. Meanwhile, some signaling pathways that would induce the resistance of antitumor drugs (EMT and KRAS) were also found to be involved in HS. These might partially explain why the patients in HS had worse pathological features and prognosis.

### Tumor Immune Microenvironment and the Expression of Different Molecules

Previous studies have found that the infiltration of types of immune cells was significantly associated with the clinical outcomes of CRC patients (Galon et al., 2006; Craig et al., 2020; Picard et al., 2020). Based on the previous analyses, we found that the OS between NS and HS were statistically different. We wondered whether it was also associated with different TIME between the two subgroups. Then, we analyzed the immune-cell infiltration of each CC sample in both the training and validation sets using CIBERSORT.

**FIGURE 7:**
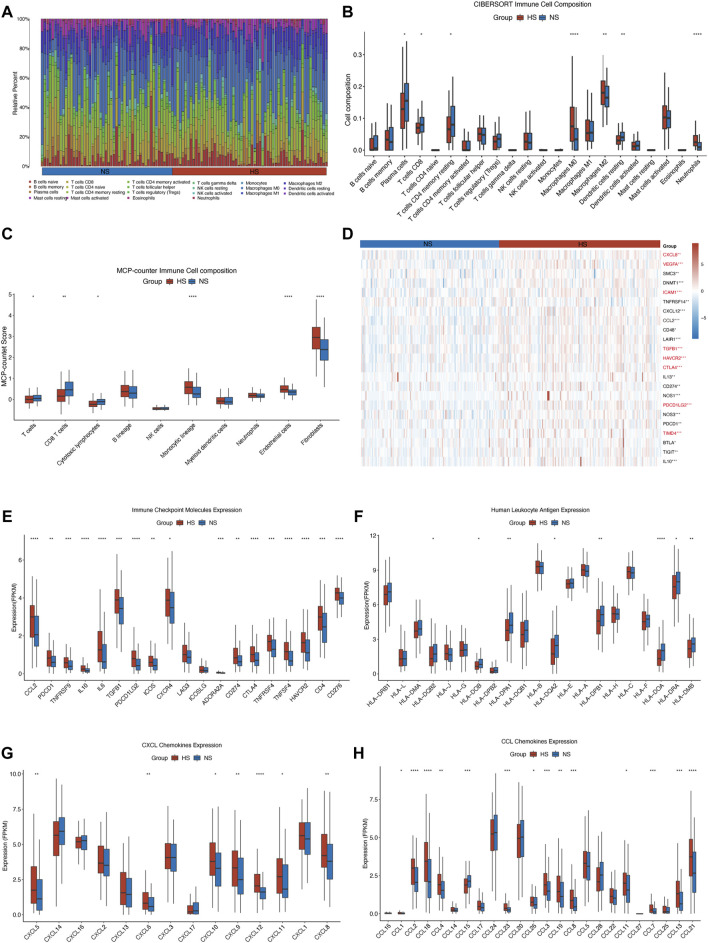
Illustration of different infiltration of immune cells and expression of immune checkpoint molecules and chemokines in NS and HS. In the TCGA-COAD cohort, the stacked bar chart **(A)** and the grouped bar chart of CIBERSORT analysis **(B)** showed that a higher level of anti-inflammation macrophages (M0 and M2) and neutrophils and a lower level of T cell (especially CD8+ T cell) were in the HS. The result of MCP-counter **(C)** confirmed that T cells, CD8+ T cells, and cytotoxic lymphocytes were enriched in NS, compared with those in HS. It also indicated that the infiltration of endothelial cells and fibroblasts was higher in HS. **(D)** The heatmap displayed the expression of negatively regulatory genes in different subgroups. The immune checkpoint genes were marked in red font. Almost all of the immune checkpoint genes **(E)**, CXCL **(G)**, and CCL **(H)** chemokines were highly expressed in HS. But some human leukocyte antigens **(F)**, including HLA-DQB2, HLA-DOB, HLA-DPA1, HLA-DQA2, HLA-DPB1, HLA-DOA, HLA-DRA, and HLA-DMB, were lowly expressed in HS. * represents p < 0.05, ** represents p < 0.01, *** represents p < 0.001, **** represents p < 0.0001.

The result showed that the HS had a significantly smaller number of CD8^+^ T and resting memory CD4^+^ T cells and a larger number of M0 macrophages in the training (Figures 7A,B) and validation (Supplementary Figure S2A) sets. While in the training set, we could also find that the number of plasma and resting dendritic cells was smaller in the HS than that in the NS. Compared with that, HS had a higher infiltration level of M2 macrophages and neutrophils (Figure 7B). Moreover, to ensure the stability of the results, we then used another algorithm (MCP-counter) to evaluate the infiltration of immune cells in different subgroups. The results showed that the infiltration of T cells, CD8^+^ T cells, and cytotoxic lymphocytes was significantly fewer in HS, whereas the infiltration of monocytic cells, endothelial cells, and fibroblasts was enriched in HS (Figure 7C). These results revealed that in the hypoxic microenvironment, the infiltration of pro-inflammation immune cells was significantly impeded, whereas the anti-inflammation immune cells and fibroblast were enriched in the hypoxic conditions that would also hinder the proliferation and migration of antitumor immune cells (Vitale et al., 2019; Davidson et al., 2021).

We then investigated the differential expression of negative regulatory immune-related genes, immune checkpoint molecules, human leukocyte antigen, CXCL, and CCL chemokines. First, we found that numerous negative regulator immune-related genes, including some immune checkpoint genes, were highly expressed in HS (Figure 7D). Second, we also found that almost all of the immune checkpoint molecules were highly expressed in HS, except LAG3 and ICOSLG (Figure 7E). Multiple studies also found that the highly expressed immune checkpoint molecules would inhibit the antitumor immunity, resulting in a poor prognosis for colorectal cancer patients (Neupane et al., 2021; Kudo-Saito et al., 2021; Gordon et al., 2017). Besides, the expression of some human leukocyte antigens (HLAs) was lowly expressed in HS, which was also consistent with the results of fewer infiltration of pro-inflammation immune cells in HS (Figure 7F). A plenty of studies have confirmed that impediments to processing HLA would hinder the identification, migration, and infiltration ability of tumor-infiltrating lymphocytes (TILs), thus facilitating the proliferation and invasion of malignancy (Dong et al., 2021; Maggs et al., 2021; Kawazu et al., 2022).

Next, we found that most chemokines, including CXCL5, 6, 8, 9, 10, 11, and 12, CCL1, 2, 3, 4, 7, 8, 11, 12, 13, 19, 21, 23, 26, and 28, were significantly highly expressed in HS (Figures 7G,H). Other studies revealed that the upregulated expression of chemokines, including CXC and CC chemokine families, could impede the TILs infiltrating into a tumor, support the growth of malignant cells, and facilitate the migration of myeloid-derived suppressor cells (MDSCs), which would cause the drug resistance to the chemotherapy and immunotherapy (Korbecki et al., 2020; Bullock and Richmond, 2021; Matsuo et al., 2021).

All in all, these findings revealed that the TIME and the expression of immune checkpoint molecules, immune-related genes, and chemokines were quite different between NS and HS, which might be associated with different pathological features and prognosis between the two subgroups. Next, based on the findings mentioned above, we would evaluate the differences in therapeutic responses between NS and HS.

### Drug Response of Immune Checkpoint Blockade, Cytotoxic, and Targeted Medicine

We used several methods to evaluate the therapeutic responses between NS and HS, including the website tool (ImmuCellAI: http://bioinfo.life.hust.edu.cn/ImmuCellAI#!/) for ICB and *pRRophetic* package for targeted and cytotoxic medicine.

**FIGURE 8:**
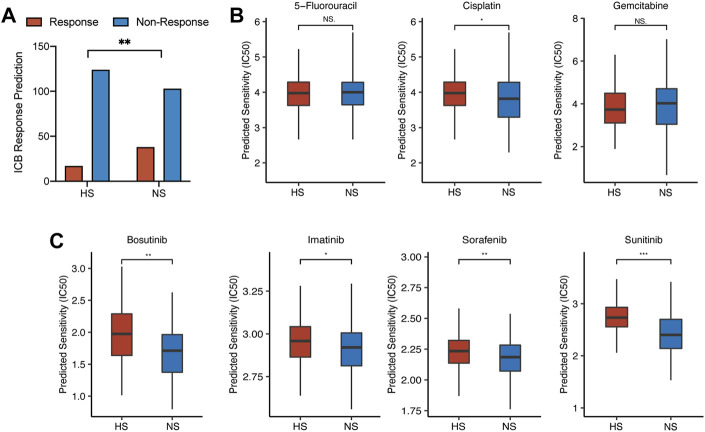
The differences in drug response between NS and HS. **(A)** ICB response prediction showed that the number of patients sensitive to ICB therapy was larger in NS than that in HS (38 in NS vs. 17 in HS). **(B)** The results of cytotoxic therapy response prediction revealed that the IC50s of these drugs were comparable between the two subgroups. **(C)** The IC50s of targeted medicine, including bosutinib, imatinib, sorafenib, and sunitinib, were lower in NS, compared with those in HS.

First, in NS, there were 38 CC (26.9% of all CC patients in NS) patients who responded to ICB therapy. Compared with that, only 17 CC (12.1% of all CC patients in HS) patients would benefit from ICB therapy. The difference between the two subgroups was statistically significant (*p* = 0.002) (Figure 8A). Second, we evaluated three cytotoxic drugs that were most widely used in clinical application, including 5-fluorouracil (5-FU), cisplatin, and gemcitabine. The result showed that the half inhibitory concentration (IC50) of cisplatin was statistically lower in NS (Figure 8B). For targeted medicine, we found that the IC50s of bosutinib, imatinib, sorafenib, and sunitinib were all lower in NS (Figure 8C).

Although most targeted drugs were not approved to be used as the first-line therapy for CRC by FDA, some studies revealed that tyrosine kinase inhibitor (TKI), including bosutinib, imatinib, and sunitinib, could enhance the infiltration of cytotoxic and effector T cells, which would directly affect the efficacy of immunotherapy (Roulleaux Dugage et al., 2021; Tazzari et al., 2021; Hirata et al., 2022). These studies revealed that the application of TKI might be positively related to the infiltration of pro-inflammation immune cells, which meant that the combination therapy of TKI and ICB might receive better efficacy than monotherapy. Recently, in a CRC mouse model, researchers found that the combination therapy of TKI and ICB could reduce tumor-stromal volume and increase the infiltration of CD8^+^ T cells and the activation of immune-related pathways (Yorita et al., 2021).

### Analyses of Correlated Expression and Quantitative Real-Time PCR for Hub Genes

**FIGURE 9:**
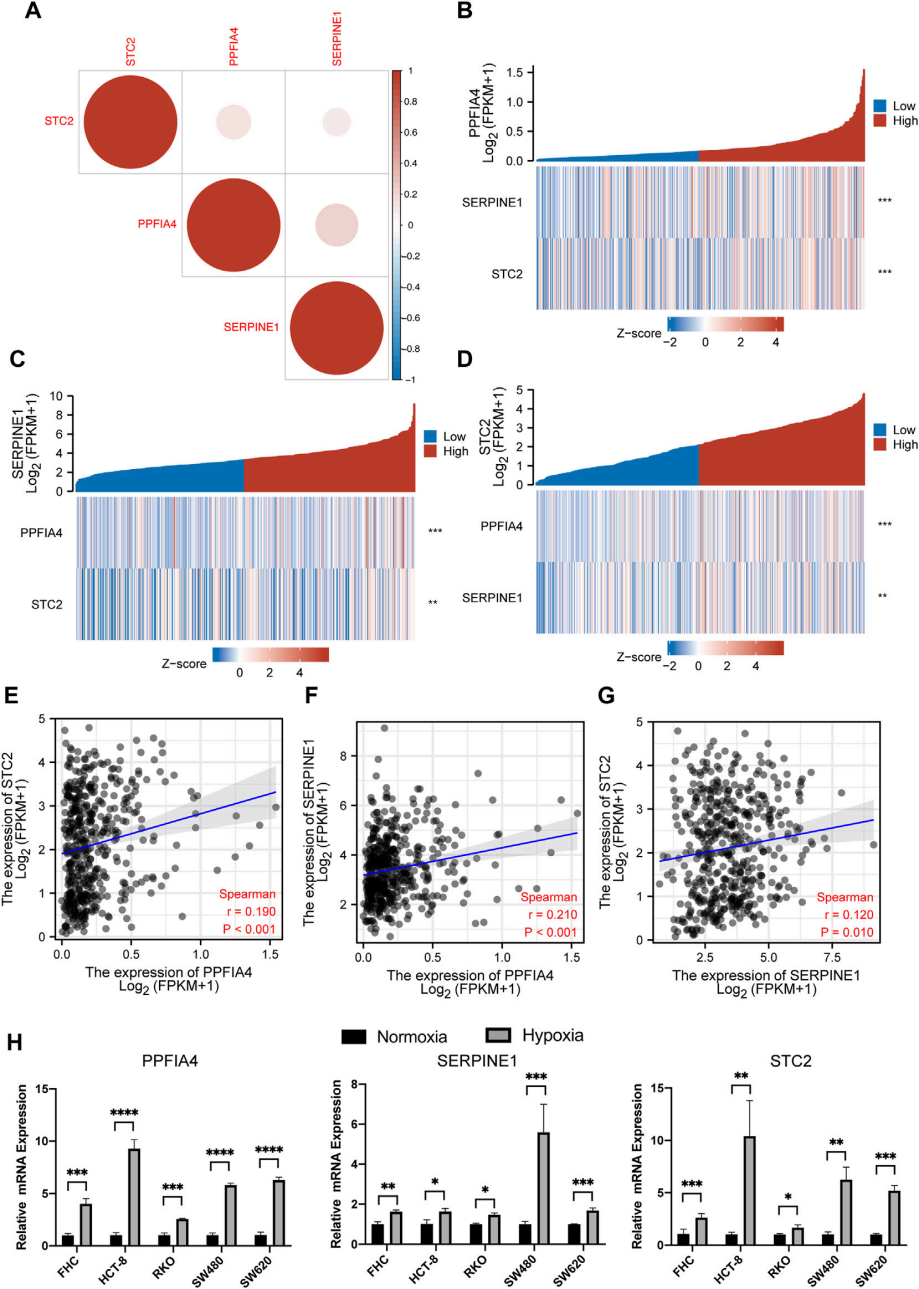
The correlation among the expression of three hub genes. **(A)** The plot illustrated that the expression of all three genes was positively related. **(B,C,D)** Three expression correlation plots visualized that with increasing expression of one hub gene, the expression of two other hub genes statistically increased. **(E,F,G)** The plots of the Pearson correlation coefficient showed the correlation between PPFIA4 and SERPINE1 was the strongest, followed by the correlation of PPFIA4 and STC2 and the correlation between SERPINE1 and STC2. This result was following the previous result shown in **(A)**. The results of qPCR **(H)** showed that three hub genes were highly expressed under hypoxic cultivation in different colon cell lines, including colon epithelial cell line (FHC) and CRC cell lines (HCT-8, RKO, SW480, and SW620). * represents p < 0.05, ** represents p < 0.01, *** represents p < 0.001, **** represents p < 0.0001.

Although our previous work revealed that the expression of three hub genes (PPFIA4, SERPINE1, and STC2) might be positively related, we still wondered whether there were direct correlations among these genes. The results suggested that all three genes were indeed positively expressed (Figure 9A). Meanwhile, we found that the correlation between the expression of PPFIA4 and SERPINE1 was statistically highest (Figures 9B–D). To ensure the accuracy of the results, we also used the website tool TIMER to evaluate the correlations among three hub genes. The results confirmed that these genes were statistically positively related (Figures 9E–G).

All 200 genes included in our analysis were in the hallmark hypoxia gene set of the GSEA database. Next, we used qPCR to confirm whether three hub genes were highly expressed in the hypoxic conditions. We used five types of colon cell lines, including 1 colon epithelial cell line (FHC) and 4 CRC cell lines (HCT-8, RKO, SW480, and SW620). Among them, HCT-8 and RKO were dMMR/MSI cell lines, while SW480 and SW620 were pMMR/MSS. For the hypoxic conditions, cell lines were cultivated in a hypoxia incubator with 1% O_2_ and 5% CO_2_ for 24 h. The result showed that all three hub genes were statistically highly expressed in the hypoxic conditions (Figure 9H).

### Analysis of Each Hub Gene

All previous analyses were about the risk score model that was constructed with hub genes and related coefficients. We then analyzed the correlation of the expression of single hub gene with clinicopathological features and immune infiltration (Supplementary Figures S3–5).

The results suggested that a higher level of expression of all three hub genes was found in the tumor samples and was associated with a poorer prognosis for CC patients. Meanwhile, all three genes were positively related to the infiltration of macrophages, neutrophils, and NK cells. However, PPFIA4 and STC2 were negatively associated with CD8^+^ T cells, T cells, and cytotoxic cells, which were following the previous results of TIME in different subgroups that CC patients with higher risk scores had a lower level of infiltration of CD8^+^ T cells (Figure 7).

### Multiple Immunohistochemistry Staining

Finally, we wanted to validate the results of the CIBERSORT analysis, which suggested that the expression of hub hypoxia-related genes (PPFIA4, SERPINE1, and STC2) was associated with the infiltration of some immune cells. We collected 35 colon cancer tissue samples from the Department of General Surgery, Changzhou Wujin Hospital. All the samples were fixed by formalin and embedded in paraffin.

**FIGURE 10:**
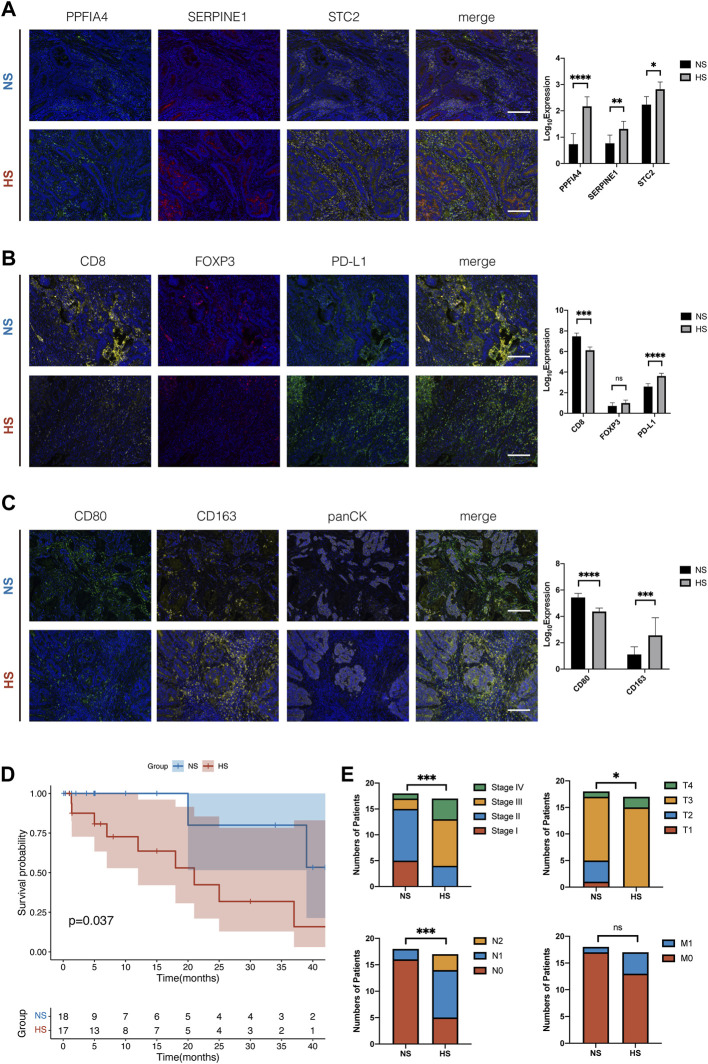
Multiple immunohistochemistry staining of tissue samples from 35 colon cancer patients. **(A)** Typical figures of the expression of three hub hypoxia-related genes, including PPFIA4, SERPINE1, and STC2, in NS and HS. According to the median of expression, we divided 35 CC patients into NS and HS. The histogram showed that the expression of three genes was significantly different, and three genes were positively related. **(B)** Compared with NS, HS had smaller colonization of CD8+ T cells and higher expression of PD-L1. But the number of Treg cells (FOXP3) had no statistical differences between subgroups. **(C)** The colonization of M1 macrophages was larger and M2 macrophages was smaller in NS, compared with that in HS. **(D)** The survival plot of K-M analysis of the 35 colon cancer patients. **(E)** The differences of the pathological features, including pathological stage (Stage), invasion depth (T), lymphocytic metastasis (N), and distant metastasis (M), of the 35 colon cancer patients. * represents *p* < 0.05, ** represents *p* < 0.01, *** represents *p* < 0.001, **** represents p < 0.0001.

The results showed that the expression of three hub genes was positively related (Figure 10A). NS had a relatively lower expression of hypoxia-related genes. We divided 35 samples into a lower expression subgroup (NS: 18) and a higher expression subgroup (HS: 17), according to the median of the number of positively expressed cells. Then, we checked their immune status, including CD8^+^ T cells, Treg cells, the expression of PD-L1, M1, and M2 macrophages. In Figures 10B,C, the results suggested that the NS had larger colonization of CD8^+^ T cells and M1 macrophages (CD80) than HS, which was insistence with our previous results in Figures 7A,B. Meanwhile, similar to the results shown in Figures 7B,E, M2 macrophages (CD163) and PD-L1 were statistically less in NS, compared with those in HS. Moreover, we also compared the pathological feature between NS and HS of the 35 colon cancer patients. The results showed that the OS and pathological variates, including pathological stage, invasion depth, and lymphocytic metastasis, were significantly better than those of the HS (Figures 10D,E).

Combined with these results, we identified that NS, which represented normoxia TIME, had relatively “hot” TIME. It had enriched colonization of pro-inflammation immune cells (CD8^+^ T cells, M1 macrophages), less population of anti-inflammation immune cells (M2 macrophages), and higher expression of immune checkpoint molecules (PD-L1). Compared with HS, the TIME of NS would be more suitable for TILs to survive and exhibit their tumor-killing function. On the other hand, the results of mIHC were similar to those analyzed by CIBERSORT, indicating its stable performance.

## Discussion

In the present study, we have identified a hypoxia-related gene signature, which contained three hub genes, to classify CC patients into NS and HS, and subsequently predicted the different TIME and prognosis between different subgroups. Based on this signature, a risk score model was constructed and proven to be a valuable and stable prognostic tool for CC patients. Moreover, we identified that the TIME and the gene expression were quite different, which might induce distinct drug responses between NS and HS. Based on ImmuCellAI and *pRRophetic* package, we found that patients in NS were more sensitive to ICB and targeted therapies. Finally, three hub genes (PPFIA4, SERPINE1, and STC2) were confirmed to be highly expressed in the hypoxic conditions by qPCR. Also, the infiltrations of CD8^+^ T cells and M1 macrophages were proven to be negatively related to the expression of these genes by mIHC.

Our present work identified that hypoxia-related genes and the risk score model built on them were significantly correlated with the prognosis of CC patients. Similar results were found in ovarian cancer (Chen et al., 2021), triple-negative breast cancer (Yang et al., 2021a), osteosarcoma in children (Jiang et al., 2021a), acute myeloid leukemia (Jiang et al., 2021b), and so on. It indicated that along with other factors, hypoxia might play an important role in the development and progression of cancer.

In our risk score model, three hub genes, including PPFIA4, SERPINE1, and STC2, were all identified to be differentially expressed between normal and tumor tissues and be closely associated with the prognosis and infiltration of immune cells in CC patients. PPFIA4, belonging to the PPFIA family of kinesin-cargo linkers, was first identified and characterized in silico in 2003 (Katoh and Katoh, 2003). Recently, it has been proved to be related to CC cell proliferation and migration by enhancing tumor glycolysis (Huang et al., 2021). Besides, previous studies suggested that PPFIA4 was also the key prognostic gene in thyroid and prostate cancer (Xu et al., 2021a; Xu et al., 2021b). Compared with PPFIA4, the role of SERPINE1 in CC had been more deeply investigated. Some studies found that the expression of SERPINE1 was negatively associated with tumor grade and response to adjuvant therapy of CC patients (Halamkova et al., 2011; Cheng et al., 2018). Meanwhile, SERPINE1 has also been proven to play an important role in remodeling TME and enhancing tumor progression in CC (Wang et al., 2021). For STC2, it was recognized as a regulator in CC cell biological processes, and silencing STC2 could effectively suppress cancer cell proliferation, survival, and migration (Li et al., 2019). Moreover, higher expression of STC2 mRNA in tumor tissues was correlated with larger tumor size, presence of venous invasion, lymphatic invasion, distant metastasis, and poorer prognosis of CC patients (Watanabe et al., 2021). However, many studies we mentioned above were only based on the bioinformatics analyses, which were not stable and persuasive enough. Therefore, besides bioinformatics analyses, we also used cell lines and tumor tissues to perform qPCR and mIHC for validation.

The limit of nutrients and oxygen, which also restrains the proliferation of tumor cells, stimulates tumors to enhance the growth of new vasculatures. However, these newly formed vessels are leaky for their discontinuous endothelium, which will induce high permeability and permeation (Majidpoor and Mortezaee, 2021). The disorganized vasculatures, along with the high level of metabolic rate and the low efficiency of ATP producing method of tumor cells, cause a severe hypoxic condition in TME (Bertout et al., 2008). In addition, the hypoxia TME simultaneously promotes the famous “Warburg effect,” enhancing glycolysis and lactic acid production catalyzed by the lactate dehydrogenase A (LDH-A) (Harris, 2002). Subsequently, it will result in acidic pH, which impairs cytotoxicity and proliferation of types of immune cells by reducing their chemotaxis, respiratory activity, and bactericidal ability (Jing et al., 2019). Taken together, the hypoxia and acidic TME greatly suppress the antitumor immune function and thus induce tumor survival and metastasis. Among numerous immune cells infiltrating in TME, macrophages are the principal component, which can differentiate into the tumor-associated macrophages (TAMs) that have been identified to be preferentially located in almost all tumor hypoxic regions (Hegde et al., 2021). Different from M1 TAMs, M2 TAMs play an anti-inflammatory role in the TME by secreting immunosuppressive molecules, including IL-10, human leukocyte antigen G (HLA-G), TGF-β (Komohara et al., 2016). They directly with MDSCs restrain the infiltration of antitumor T cells and their secretion of IFN-γ. In the present study, less infiltration of CD8^+^ T cells and more infiltration of M2 macrophages were found in the high-risk group in both bioinformatics and mIHC analyses. Meanwhile, M2 TAMs were proven to express increased programmed cell death ligand 1 (PD-L1). It has been established that increased expression of PD-L1 was related to poor prognosis in numerous malignancies (Pérez-Ruiz et al., 2020). Accordant with the previous study, we also found that the expression of immune checkpoint molecules was higher in the high-risk group, which indicated that they might be associated with hypoxia condition. A recent study proved that tumor cells might escape immune attacks from both innate and adaptive immune systems by secreting hypoxia-inducible factor 1 (HIF-1) (You et al., 2021). Since suppressive TAMs, immune checkpoint molecules, and HIF-1 were all important negative factors, blocking them has predictably received promising results in enhancing the infiltration of tumor-infiltrating lymphocytes (TILs), thus improving their tumor-killing effect (Yang et al., 2021b; Caushi et al., 2021; Yap et al., 2021; You et al., 2021).

Based on the knowledge that hypoxia was a key barrier to antitumor immunity, studies have focused on how to target the hypoxic metabolic production or reverse the hypoxic condition in TME. First, enhancing tumor oxygenation is an option, which could be applied by carbogen breathing and intervention to reduce O_2_ consumption by the tumor (Kheir et al., 2012; Zou et al., 2018). Second, hypoxia-activated prodrugs (HAPs) were designed to specifically target those hypoxic tumor cells. These HAPs could bring both genotoxic agents and non-genotoxic effectors (Penketh et al., 2012; Skwarska et al., 2021). Third, targeting acidosis or hypoxia-acidosis-related pathways in TME is another choice (Singleton et al., 2021). Moreover, the combination therapy could also enhance the antitumor immunity and reduce drug toxicity and resistance. For instance, the objective response rates (ORR) of monotherapy of PD-1 and CTLA-4 blockers were 10%–16% for ipilimumab and 10%–40% for nivolumab and pembrolizumab (Robert et al., 2011; Ribas et al., 2015; Robert et al., 2015). While the combination of them could significantly increase the ORR to 61% and reduce the incidence of grade 3–4 adverse events to 46% (Long et al., 2017). The combination of ICBs with chemotherapy, targeted therapy, radiation, or intratumoral therapy also showed gratifying therapeutic results in treating different types of malignancies (Meric-Bernstam et al., 2021). The reason is these therapeutic methods could enhance the activation and infiltration of TILs by inhibiting angiogenesis, normalizing vasculature, reconstructing immunosupportive TME, increasing antigen presentation, co-stimulating molecules, and so on.

Recently, thanks to the advance in bioinformatics, we have deeper insight into the genomics of human beings. The function of more gene signatures has been identified. Using the existing gene signature, scientists can filter out hub genes and build risk models that have prognostic power and can depict different tumor microenvironments of different cancer patients.

These risk models were built using different statistical methods. For instance, Guan et al. (2020) established a prediction model that could separate gastric cancer patients into two different subgroups using the immune-related gene signature (Guan et al., 2020). They used the ssGSEA score and the hierarchical clustering algorithm. One subgroup had a higher expression of the immune-related score and better prognosis and another had a lower score and poorer prognosis. They successfully associated the tumor immune microenvironment with the prognosis in the population of gastric cancer patients. Based on the immune checkpoint-related gene signature, another study built a risk model to predict the prognosis of hepatocellular carcinoma patients using Lasso and Cox regression analyses (Zhao et al., 2020). Their model could also divide liver cancer patients into 2 subgroups, which had different prognosis and tumor immune microenvironments. The third example is that Bagaev et al. (2021) built a model, which had 29 knowledge-based functional gene signatures, to separate cancer patients into 4 subtypes, including immune-enriches (fibrotic), immune-enriched (non-fibrotic), fibrotic, and depleted (Bagaev et al., 2021). They used the ssGSEA score and the Louvain clustering method. These subtypes had a quite different infiltration of immune cells and prognosis. The most important thing is that this model is pan-cancer conserved, which means that it can be used in most types of cancer and has great clinical application potential. Like the previous studies, we also used Lasso and Cox regression analyses to identify hub genes and built a risk model subsequently. We believe that along with the advance in bioinformatics and statistics, more powerful prediction models will be developed.

There are several limitations in the present study. First, all analyses were based on retrospective data from public databases, which will induce recall and selection biases. Second, although related work to eliminate the batch effect has been done, the potential risk still exists when analyses are based on data from two different databases. Finally, our biomolecular experiments were just to verify the results of bioinformatics analyses. They have not deeply uncovered the bio- and molecular mechanism of hypoxia involved in the development and progression of CC. However, our further work will continuously focus on this field.

## Conclusion

In summary, based on the hypoxia-related genes, we constructed a risk score model to predict the prognosis of colon cancer patients. Moreover, we deeply analyzed the differences, including functional enrichment, infiltration of immune cells, expression of different genes (immune checkpoint genes, human leukocyte antigen, CXCL, and CCL chemokines), and the therapeutic responses, between high-risk and low-risk subgroups. Finally, we performed qPCR and multiple immunohistochemistry (mIHC) for validation. This stud might provide new insights into the association among hypoxia, clinical prognosis, TIME, and therapy.

## Data Availability

The original contributions presented in the study are included in the article/Supplementary Material, further inquiries can be directed to the corresponding authors.

## Supplementary Material

Supplementary Figure S1
**(A)** The calibration analysis suggested that the prognostic performance of the risk score model was stable in the validation set. **(B)** The DCA analysis showed that the prognostic ability of the risk score model plus clinical factors was best, followed by the risk score model alone and clinical factors alone. **(C)** The nomogram was built based on the risk score model and several clinical variates. **(D)** The ROC analysis of nomogram in the validation set.

Supplementary Figure S2 Analyses of TIME were also applied in the validation set. The stacked bar chart **(A)** and the grouped bar chart of CIBERSORT analysis **(B)** showed that a higher level of anti-inflammation M0 macrophages and neutrophils and a lower level of CD8^+^ T cell and CD4 memory resting cells were in the HS. **(C)** The heatmap displayed the expression of negatively regulatory genes in different subgroups. The immune checkpoint genes were marked in red font. The immune checkpoint genes, including PD-L1, HAVCR2, PD-1, CTLA4, and TGF-β1, were statistically higher expressed in HS. * represents *p* < 0.05, ** represents *p* < 0.01, *** represents *p* < 0.001, **** represents *p* < 0.0001.

Supplementary Figure S3 The correlation among the expression of PPFIA4, clinical prognosis, and infiltration of immune cells. In the TCGA database, the results in the paired **(A)** and non-paired **(B)** tissue samples showed that the expression of PPFIA4 was statistically higher in the tumor samples. The results of K-M analyses of overall **(C)** and disease-specific **(D)** suggested that the expression of PPFIA4 and clinical prognosis were statistically negatively correlated. **(E)** The Lollipop chart revealed that the expression of PPFIA4 was positively related to the infiltration of macrophages, and negatively related to the infiltration of most types of T cells. **(F)** The results of the Pearson Correlation Coefficient suggested that the expression of PPFIA4 was significantly associated with the infiltration of immune cells. * represents *p* < 0.05, ** represents *p* < 0.01, *** represents *p* < 0.001, **** represents *p* < 0.0001.

Supplementary Figure S4 The correlation among SERPINE1, clinical prognosis, and infiltration of immune cells. In the TCGA database, the results of the PCR tests in the paired **(A)** and nonpaired **(B)** tissue samples showed that the expression of SERPINE1 was statistically higher in the tumor samples. The results of K-M analyses of overall **(C)** and disease-specific **(D)** suggested that the expression of SERPINE1 and clinical prognosis were statistically negatively correlated. **(E)** The Lollipop chart revealed that the expression of SERPINE1 was positively related to the infiltration of macrophages and most types of T cells. **(F)** The results of the Pearson Correlation Coefficient suggested that the expression of SERPINE1 was significantly associated with the infiltration of immune cells. * represents *p* < 0.05, ** represents *p* < 0.01, *** represents *p* < 0.001, **** represents *p* < 0.0001.

Supplementary Figure S5 The correlation among STC2, clinical prognosis, and infiltration of immune cells. In the TCGA database, the results of PCR tests in the paired **(A)** and nonpaired **(B)** tissue samples showed that the expression of STC2 was statistically higher in the tumor samples. The results of K-M analyses of overall **(C)** and disease-specific **(D)** suggested that the expression of STC2 and clinical prognosis were statistically negatively correlated. **(E)** The Lollipop chart revealed that the expression of STC2 was positively related to the infiltration of macrophages, and negatively related to the infiltration of most types of T cells. **(F)** The results of the Pearson Correlation Coefficient suggested that the expression of STC2 was significantly associated with the infiltration of immune cells. * represents *p* < 0.05, ** represents *p* < 0.01, *** represents *p* < 0.001, **** represents *p* < 0.0001.
