# Supplementary figures and images for: Novel Hypoxia-Associated Gene Signature Depicts Tumor Immune Microenvironment and Predicts Prognosis of Colon Cancer Patients
Source: Front Genet. 2022 Jun 6;13:901734. doi: 10.3389/fgene.2022.901734 (PMC9208084; doi:10.3389/fgene.2022.901734)

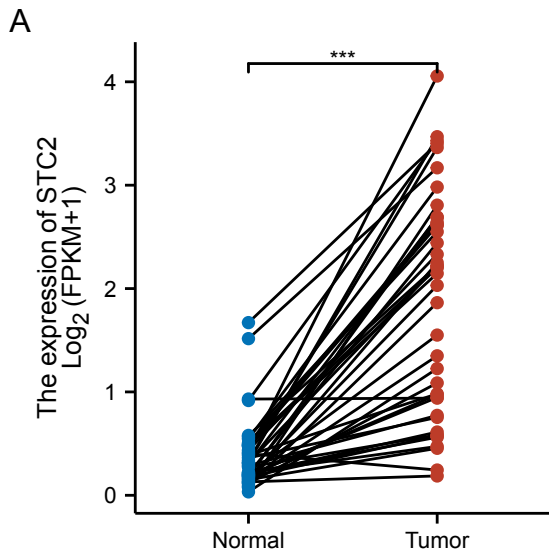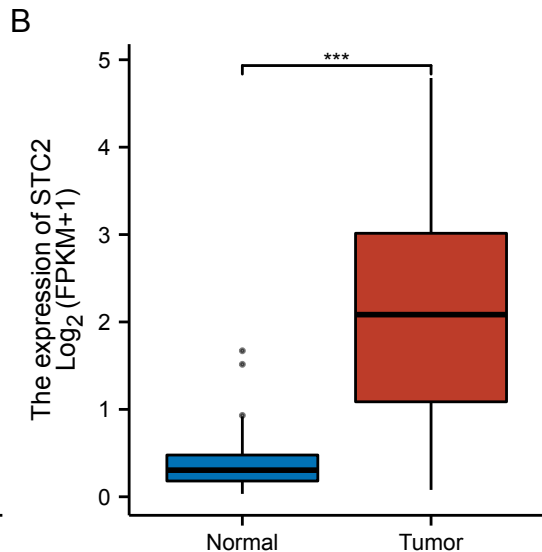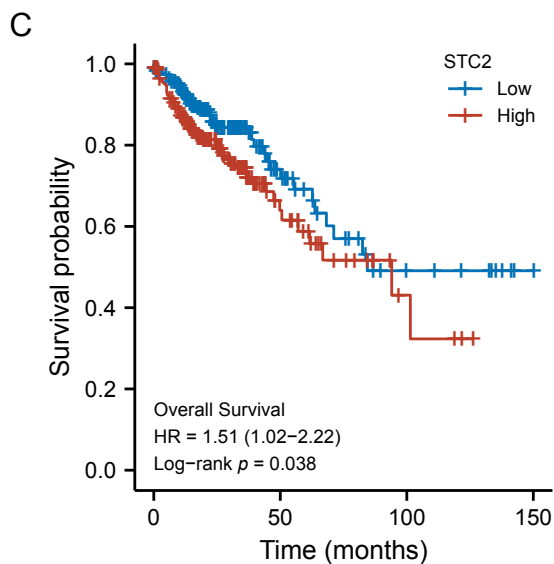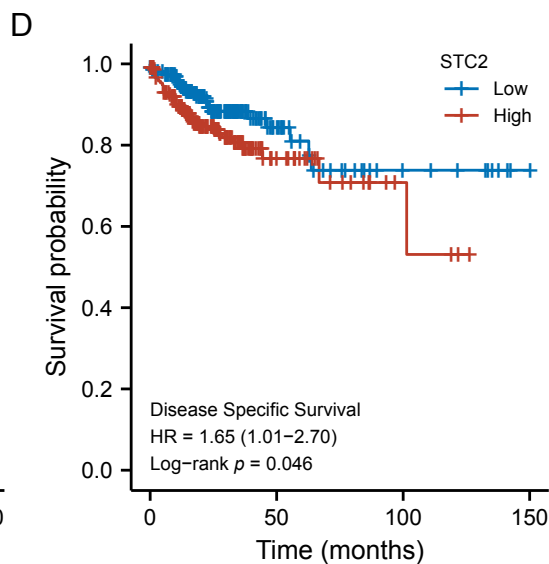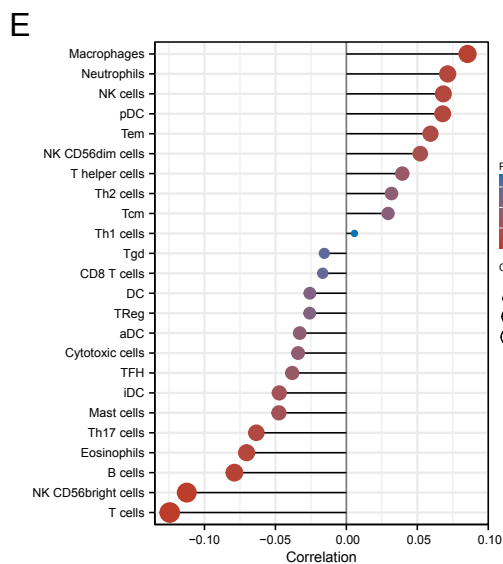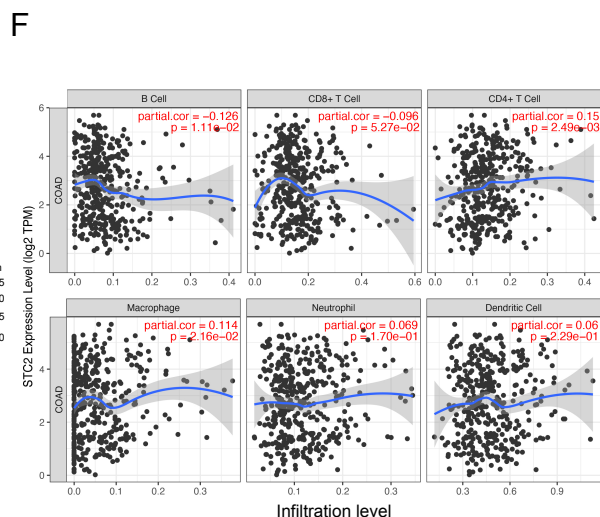

Supplement: Supplementary file 1 [file Image5.PDF]

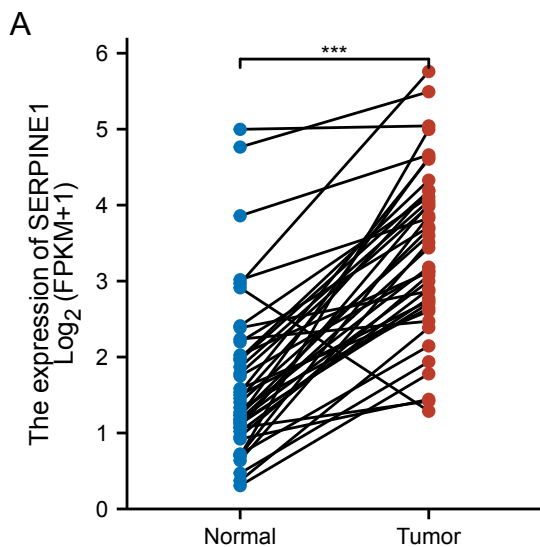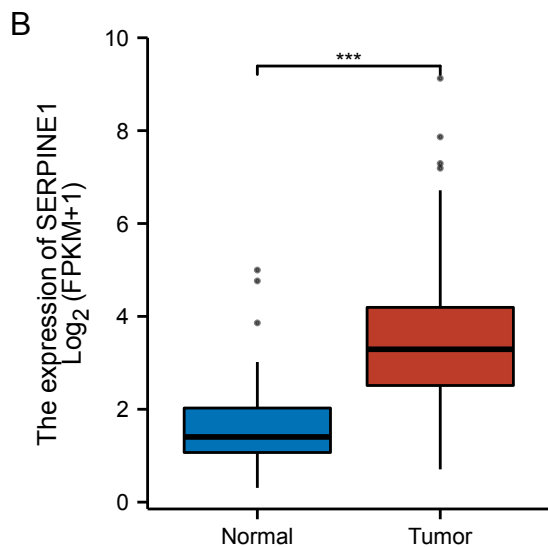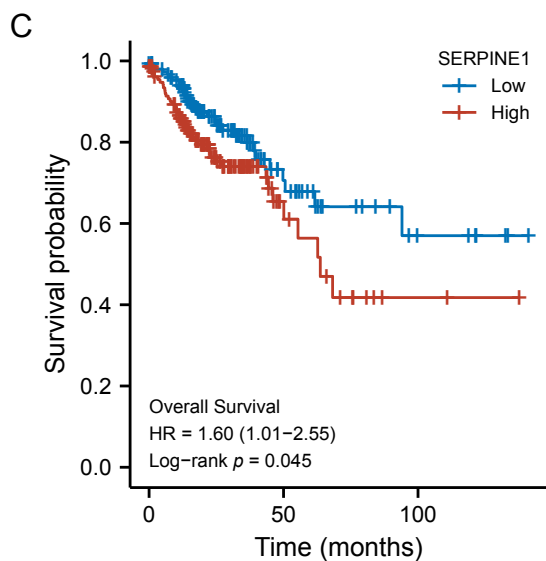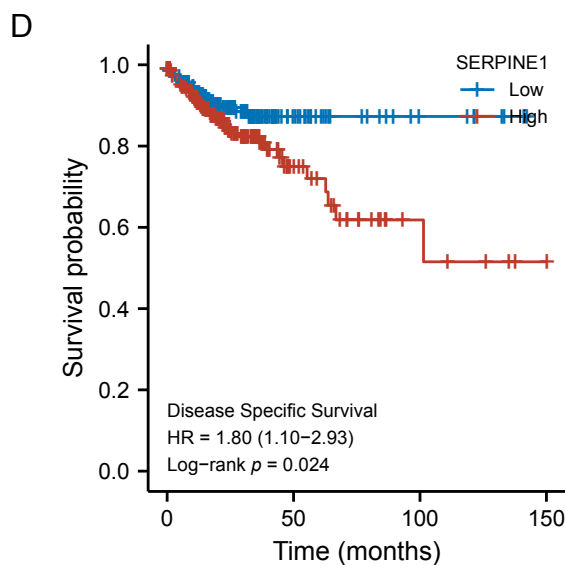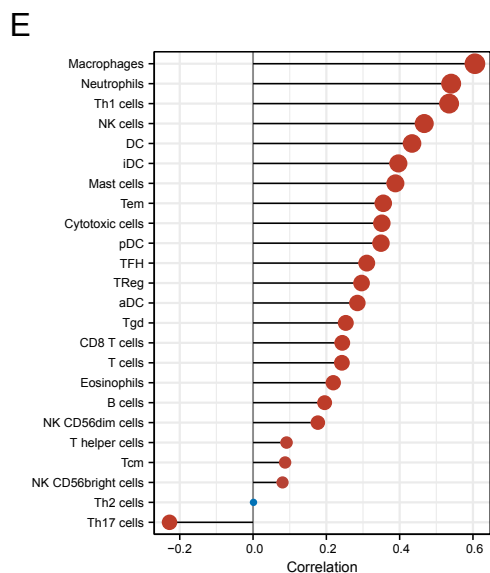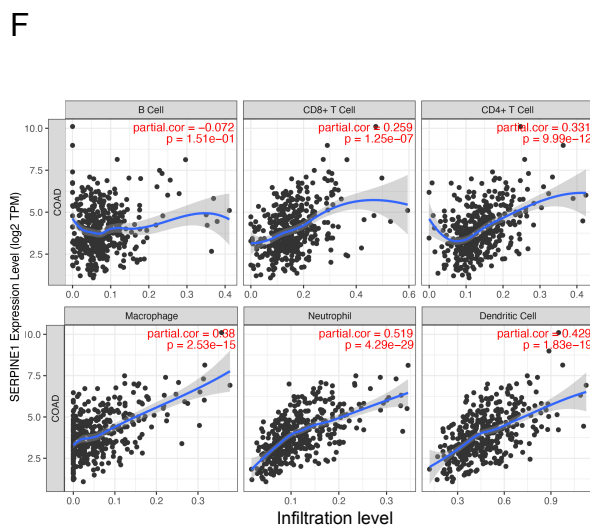

Supplement: Supplementary file 3 [file Image4.PDF]

A

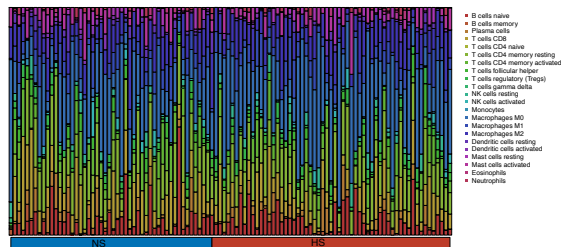

CIBERSORT Immune Cell Composition

Group ■ NS ■ HS

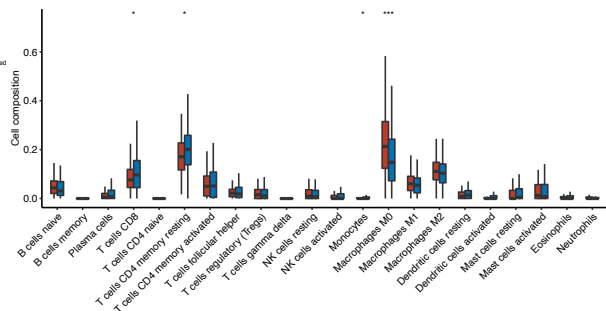

B

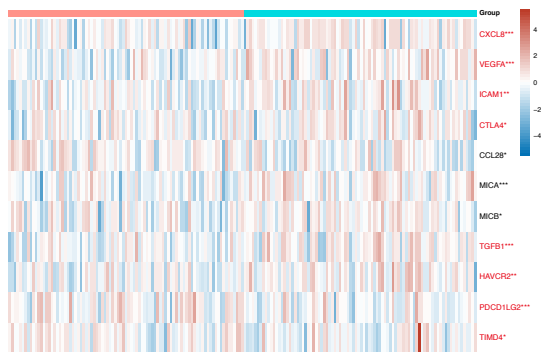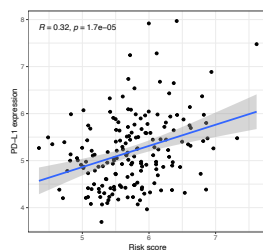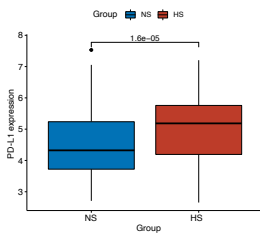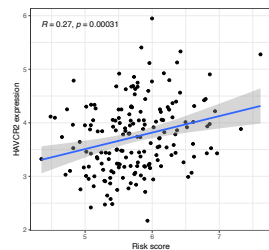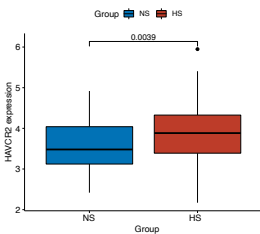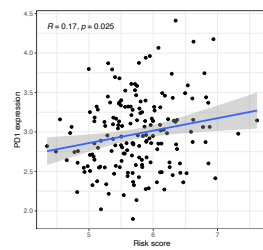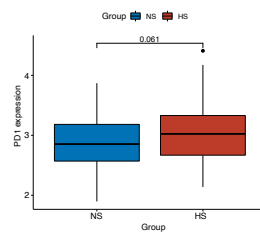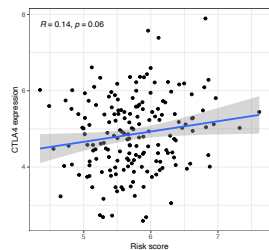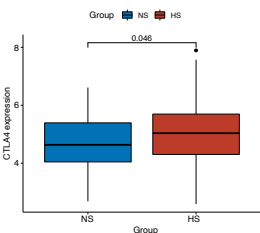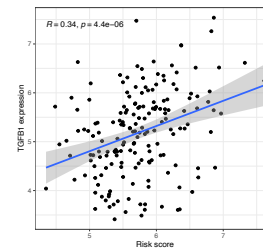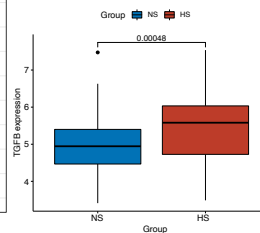

Supplement: Supplementary file 4 [file Image2.PDF]

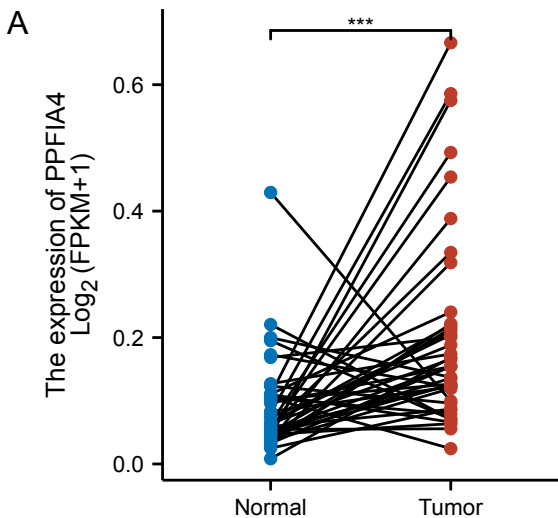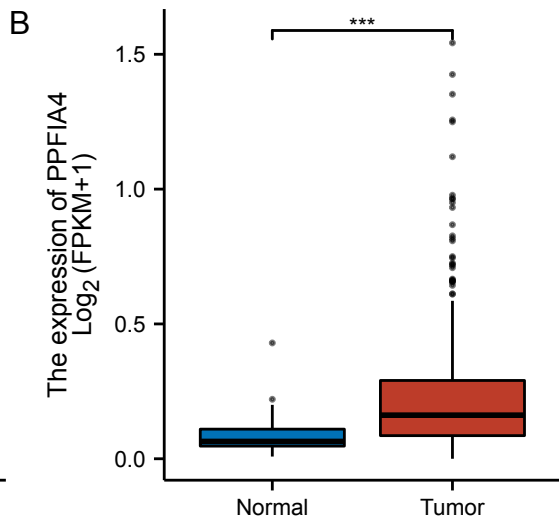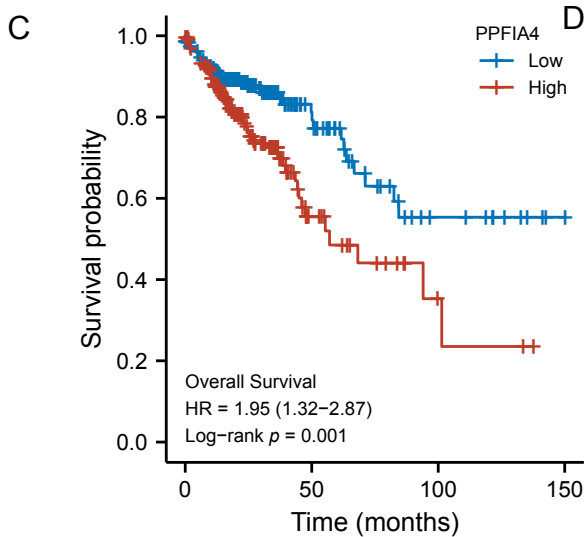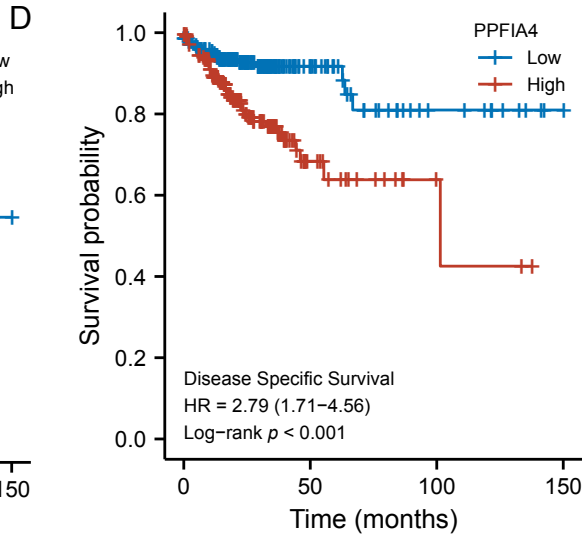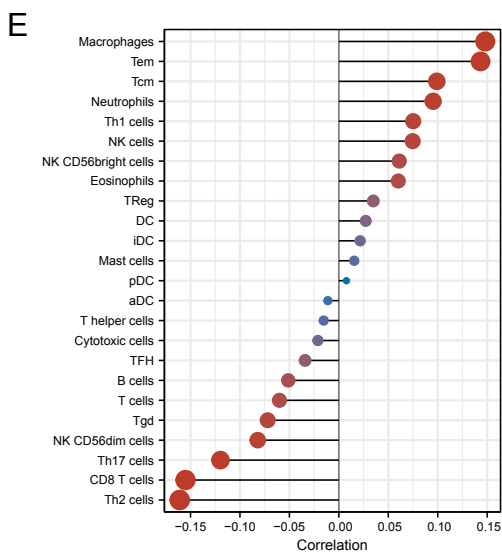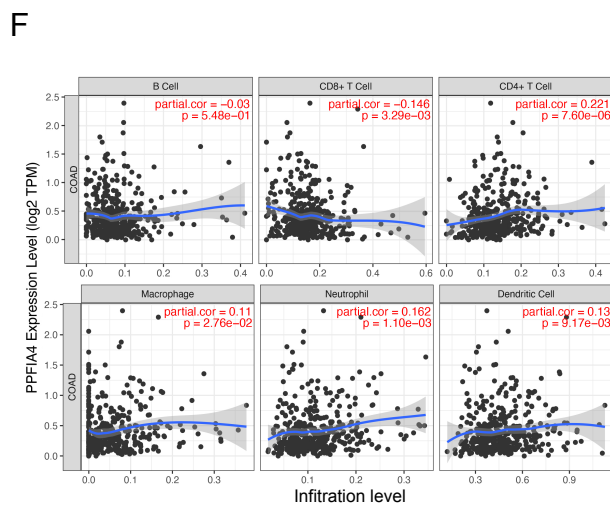

Supplement: Supplementary file 5 [file Image3.PDF]
