# Supplementary material for: Novel Hypoxia-Associated Gene Signature Depicts Tumor Immune Microenvironment and Predicts Prognosis of Colon Cancer Patients
Source: Front Genet. 2022 Jun 6;13:901734. doi: 10.3389/fgene.2022.901734 (PMC9208084; doi:10.3389/fgene.2022.901734)
Supplement: Supplementary file 2 [file Table1.DOCX]

Supplementary Table 1: The list of 200 hypoxia-related genes.

| Gene Symbol |
| --- |
| ACKR3 |
| ADM |
| ADORA2B |
| AK4 |
| AKAP12 |
| ALDOA |
| ALDOB |
| ALDOC |
| AMPD3 |
| ANGPTL4 |
| ANKZF1 |
| ANXA2 |
| ATF3 |
| ATP7A |
| B3GALT6 |
| B4GALNT2 |
| BCAN |
| BCL2 |
| BGN |
| BHLHE40 |
| BNIP3L |
| BRS3 |
| BTG1 |
| CA12 |
| CASP6 |
| CAV1 |
| CAVIN1 |
| CAVIN3 |
| CCN1 |
| CCN2 |
| CCN5 |
| CCNG2 |
| CDKN1A |
| CDKN1B |
| CDKN1C |
| CHST2 |
| CHST3 |
| CITED2 |
| COL5A1 |
| CP |
| CSRP2 |
| CXCR4 |
| DCN |
| DDIT3 |
| DDIT4 |
| DPYSL4 |
| DTNA |
| DUSP1 |
| EDN2 |
| EFNA1 |
| EFNA3 |
| EGFR |
| ENO1 |
| ENO2 |
| ENO3 |
| ERO1A |
| ERRFI1 |
| ETS1 |
| EXT1 |
| F3 |
| FAM162A |
| FBP1 |
| FOS |
| FOSL2 |
| FOXO3 |
| GAA |
| GALK1 |
| GAPDH |
| GAPDHS |
| GBE1 |
| GCK |
| GCNT2 |
| GLRX |
| GPC1 |
| GPC3 |
| GPC4 |
| GPI |
| GRHPR |
| GYS1 |
| HAS1 |
| HDLBP |
| HEXA |
| HK1 |
| HK2 |
| HMOX1 |
| HOXB9 |
| HS3ST1 |
| HSPA5 |
| IDS |
| IER3 |
| IGFBP1 |
| IGFBP3 |
| IL6 |
| ILVBL |
| INHA |
| IRS2 |
| ISG20 |
| JMJD6 |
| JUN |
| KDELR3 |
| KDM3A |
| KIF5A |
| KLF6 |
| KLF7 |
| KLHL24 |
| LALBA |
| LARGE1 |
| LDHA |
| LDHC |
| LOX |
| LXN |
| MAFF |
| MAP3K1 |
| MIF |
| MT1E |
| MT2A |
| MXI1 |
| MYH9 |
| NAGK |
| NCAN |
| NDRG1 |
| NDST1 |
| NDST2 |
| NEDD4L |
| NFIL3 |
| NOCT |
| NR3C1 |
| P4HA1 |
| P4HA2 |
| PAM |
| PCK1 |
| PDGFB |
| PDK1 |
| PDK3 |
| PFKFB3 |
| PFKL |
| PFKP |
| PGAM2 |
| PGF |
| PGK1 |
| PGM1 |
| PGM2 |
| PHKG1 |
| PIM1 |
| PKLR |
| PKP1 |
| PLAC8 |
| PLAUR |
| PLIN2 |
| PNRC1 |
| PPARGC1A |
| PPFIA4 |
| PPP1R15A |
| PPP1R3C |
| PRDX5 |
| PRKCA |
| PYGM |
| RBPJ |
| RORA |
| RRAGD |
| S100A4 |
| SAP30 |
| SCARB1 |
| SDC2 |
| SDC3 |
| SDC4 |
| SELENBP1 |
| SERPINE1 |
| SIAH2 |
| SLC25A1 |
| SLC2A1 |
| SLC2A3 |
| SLC2A5 |
| SLC37A4 |
| SLC6A6 |
| SRPX |
| STBD1 |
| STC1 |
| STC2 |
| SULT2B1 |
| TES |
| TGFB3 |
| TGFBI |
| TGM2 |
| TIPARP |
| TKTL1 |
| TMEM45A |
| TNFAIP3 |
| TPBG |
| TPD52 |
| TPI1 |
| TPST2 |
| UGP2 |
| VEGFA |
| VHL |
| VLDLR |
| WSB1 |
| XPNPEP1 |
| ZFP36 |
| ZNF292 |
