# Supplementary material for: Novel Hypoxia-Associated Gene Signature Depicts Tumor Immune Microenvironment and Predicts Prognosis of Colon Cancer Patients
Source: Front Genet. 2022 Jun 6;13:901734. doi: 10.3389/fgene.2022.901734 (PMC9208084; doi:10.3389/fgene.2022.901734)
Supplement: Supplementary file 6 [file Table2.DOCX]

Supplementary Table 2: The list of differentially expressed hypoxia-related genes between NS and HS.

| Gene | NS.Mean | HS.Mean | log_2_FC | p-value | FDR |
| --- | --- | --- | --- | --- | --- |
| BCL2 | 3.56934264 | 1.25972222 | -1.5025528 | 7.9967E-20 | 7.9967E-19 |
| CDKN1A | 108.817082 | 53.8146992 | -1.0158329 | 4.5506E-14 | 1.7428E-13 |
| VEGFA | 4.30175479 | 13.525147 | 1.65264708 | 2.4687E-22 | 5.5545E-21 |
| AKAP12 | 8.42535287 | 3.35263319 | -1.3294425 | 9.8839E-12 | 2.9901E-11 |
| ANKZF1 | 3.83780246 | 8.20485898 | 1.09619808 | 3.5869E-21 | 5.8694E-20 |
| B4GALNT2 | 21.5140803 | 1.17401607 | -4.1957571 | 2.9299E-22 | 5.8598E-21 |
| BGN | 18.7597374 | 106.994104 | 2.51181976 | 2.0053E-16 | 1.1106E-15 |
| BHLHE40 | 18.8602723 | 51.4718013 | 1.44843177 | 6.2268E-18 | 4.1512E-17 |
| CA12 | 75.3477707 | 16.1399585 | -2.222928 | 2.1614E-20 | 2.5936E-19 |
| CAV1 | 35.6738529 | 9.75666286 | -1.8704074 | 3.6787E-16 | 1.9475E-15 |
| CAVIN1 | 68.5636967 | 30.4797861 | -1.1695921 | 6.9477E-07 | 1.3593E-06 |
| CCN5 | 2.56333896 | 0.22232481 | -3.5272834 | 1.6384E-22 | 4.5372E-21 |
| CITED2 | 32.8915151 | 10.445193 | -1.6548763 | 4.553E-24 | 3.7038E-22 |
| COL5A1 | 7.6848991 | 23.5488308 | 1.61555721 | 5.2013E-11 | 1.4629E-10 |
| CP | 2.03576705 | 0.43210312 | -2.2361249 | 1.8157E-20 | 2.254E-19 |
| DCN | 44.7303641 | 13.9108947 | -1.6850393 | 9.3396E-18 | 6.004E-17 |
| DDIT4 | 16.1277917 | 46.3411746 | 1.5227457 | 2.3716E-15 | 1.0807E-14 |
| DTNA | 0.98261031 | 0.34024237 | -1.5300566 | 4.5493E-11 | 1.2998E-10 |
| DUSP1 | 103.411574 | 49.9322754 | -1.0503531 | 3.7134E-12 | 1.1625E-11 |
| EDN2 | 4.47220483 | 0.66850432 | -2.7419775 | 3.6518E-13 | 1.3016E-12 |
| EFNA3 | 3.18690207 | 12.1661418 | 1.93264513 | 5.865E-17 | 3.4055E-16 |
| ERO1A | 9.571792 | 20.6760203 | 1.11109757 | 4.1507E-16 | 2.1347E-15 |
| GALK1 | 4.28788267 | 11.5105907 | 1.42462454 | 4.1426E-18 | 2.8139E-17 |
| GCNT2 | 2.660632 | 0.21198383 | -3.6497429 | 1.1777E-24 | 3.7038E-22 |
| HK2 | 60.0048927 | 26.9690214 | -1.153777 | 3.075E-12 | 9.7106E-12 |
| HMOX1 | 35.1314284 | 11.4919828 | -1.6121345 | 2.3914E-19 | 2.0498E-18 |
| IER3 | 60.8739151 | 143.948439 | 1.24165608 | 1.4462E-10 | 3.8565E-10 |
| IGFBP1 | 0.03461702 | 0.50325127 | 3.8617254 | 0.00016931 | 0.0002709 |
| IGFBP3 | 14.666011 | 30.3721487 | 1.05027245 | 8.0783E-08 | 1.7107E-07 |
| INHA | 0.02005705 | 0.18316278 | 3.19094535 | 0.000206 | 0.00032527 |
| KDELR3 | 10.092793 | 20.3334226 | 1.0105276 | 6.8515E-11 | 1.8829E-10 |
| KIF5A | 1.14297454 | 0.22200946 | -2.3641002 | 8.5323E-19 | 6.5354E-18 |
| LARGE1 | 4.78300426 | 2.2491914 | -1.0885106 | 6.664E-21 | 9.9961E-20 |
| LOX | 1.80383525 | 3.87963294 | 1.10485258 | 0.00018073 | 0.00028789 |
| LXN | 23.9739008 | 10.22882 | -1.2288249 | 1.0594E-19 | 1.0308E-18 |
| MIF | 22.363811 | 57.4083622 | 1.36009484 | 9.5031E-16 | 4.6865E-15 |
| MT1E | 182.324262 | 35.9197504 | -2.3436573 | 9.2741E-19 | 6.9556E-18 |
| MT2A | 209.870968 | 44.6417089 | -2.2330384 | 3.9668E-20 | 4.2001E-19 |
| MXI1 | 25.3486477 | 9.24154118 | -1.4557034 | 1.0956E-22 | 3.2869E-21 |
| NDRG1 | 87.1546254 | 37.7753221 | -1.2061332 | 4.2758E-12 | 1.327E-11 |
| NEDD4L | 8.94857023 | 4.08428577 | -1.1315734 | 1.8157E-20 | 2.254E-19 |
| NR3C1 | 4.81870877 | 1.58871972 | -1.600782 | 5.9492E-21 | 9.3119E-20 |
| PCK1 | 47.4798953 | 7.05493931 | -2.7506112 | 4.8662E-19 | 3.893E-18 |
| PGF | 0.699037 | 1.89316107 | 1.43735643 | 9.133E-13 | 3.0164E-12 |
| PGM1 | 44.7984759 | 17.5607315 | -1.3510967 | 2.3739E-22 | 5.5545E-21 |
| PKP1 | 0.13828963 | 2.17461906 | 3.9749978 | 1.5443E-18 | 1.1346E-17 |
| PLAC8 | 87.2345645 | 11.1357233 | -2.9697046 | 9.6049E-23 | 3.1434E-21 |
| PPARGC1A | 3.84137626 | 1.21646399 | -1.6589297 | 1.5456E-19 | 1.4642E-18 |
| PPFIA4 | 0.06579286 | 0.16918964 | 1.36263823 | 1.3963E-07 | 2.8561E-07 |
| PPP1R3C | 3.8313998 | 0.87896675 | -2.1239911 | 6.0165E-13 | 2.1235E-12 |
| PYGM | 2.68082207 | 0.21776932 | -3.6218028 | 7.3703E-21 | 1.0613E-19 |
| SCARB1 | 8.31950536 | 17.3374722 | 1.05932391 | 8.1811E-15 | 3.4649E-14 |
| SELENBP1 | 360.714177 | 99.1049812 | -1.8638266 | 3.0414E-17 | 1.8878E-16 |
| SERPINE1 | 3.05515415 | 14.7084672 | 2.26732983 | 3.7887E-15 | 1.7049E-14 |
| SLC2A1 | 16.2004922 | 61.1132576 | 1.91544774 | 2.6148E-16 | 1.4263E-15 |
| SLC2A5 | 2.45818452 | 0.87101997 | -1.4968155 | 8.1952E-14 | 3.0415E-13 |
| SLC6A6 | 2.12715692 | 17.1199487 | 3.00868001 | 2.2313E-23 | 8.9252E-22 |
| SRPX | 15.0206637 | 2.39060036 | -2.6515037 | 1.4326E-21 | 2.5786E-20 |
| STBD1 | 3.53564987 | 1.02842894 | -1.7815333 | 5.1442E-24 | 3.7038E-22 |
| STC1 | 0.78518077 | 3.78550612 | 2.26938946 | 3.3309E-17 | 2.0324E-16 |
| STC2 | 0.35096026 | 5.01649222 | 3.83729934 | 3.5934E-20 | 3.9201E-19 |
| SULT2B1 | 1.21231905 | 10.1709577 | 3.0686142 | 3.2954E-20 | 3.7073E-19 |
| TGFBI | 12.9582662 | 135.243001 | 3.38360933 | 2.7261E-23 | 9.814E-22 |
| UGP2 | 59.9238479 | 15.5163238 | -1.9493435 | 1.288E-23 | 6.6241E-22 |

Note: DEGs were defined as |log_2_FC|>1 and FDR<0.05.
