# Supplementary material for: Novel Hypoxia-Associated Gene Signature Depicts Tumor Immune Microenvironment and Predicts Prognosis of Colon Cancer Patients
Source: Front Genet. 2022 Jun 6;13:901734. doi: 10.3389/fgene.2022.901734 (PMC9208084; doi:10.3389/fgene.2022.901734)
Supplement: Supplementary file 7 [file Table5.DOCX]

Supplementary Table 5: Antibodies used in this study.

| **Antibody** | **Catalog number** | **Concentration** | **Company** |
| --- | --- | --- | --- |
| CD8α(C8/144B) Mouse mAb (IHC Specific) | 70306 | 1:1200 | Cell Signaling Technology |
| FoxP3 (D2W8E™) Rabbit mAb (IHC Specific) | 98377 | 1:500 | Cell Signaling Technology |
| PD-L1 (E1L3N®) XP® Rabbit mAb | 13684 | 1:800 | Cell Signaling Technology |
| CD163-Polyclonal Antibody | 16646-1-AP | 1:6000 | Proteintech, China |
| CD80/B7-1-Monoclonal Antibody | 66406-1-Ig | 1:1000 | Proteintech, China |
| Pan-Keratin-Polyclonal Antibody | 26411-1-AP | 1:8000 | Proteintech, China |
| Anti-PPFIA4 Antibody | HPA054132 | 1:400 | ATLAS Antibodies |
| Stanniocalcin 2-Polyclonal Antibody | 10314-1-AP | 1:800 | Proteintech, China |
| PAI-1-Polyclonal Antibody | 13801-1-AP | 1:800 | Proteintech, China |
