# Supplementary material for: Novel Hypoxia-Associated Gene Signature Depicts Tumor Immune Microenvironment and Predicts Prognosis of Colon Cancer Patients
Source: Front Genet. 2022 Jun 6;13:901734. doi: 10.3389/fgene.2022.901734 (PMC9208084; doi:10.3389/fgene.2022.901734)
Supplement: Supplementary file 8 [file Table3.DOCX]

Supplementary Table 3: Univariate Cox analysis of hypoxia-related genes regarding the overall survival of TCGA COAD cohort.

| Gene | HR | HR.95L | HR.95H | p-value |
| --- | --- | --- | --- | --- |
| P4HA1 | 1.65758387 | 1.03932585 | 2.64362161 | 0.03384427 |
| SERPINE1 | 1.32283571 | 1.01426756 | 1.72527879 | 0.0389702 |
| CSRP2 | 1.93010268 | 1.07115992 | 3.47781529 | 0.02861307 |
| IL6 | 1.39336091 | 1.00398797 | 1.93374292 | 0.04728197 |
| STC2 | 1.37340745 | 1.0026729 | 1.88121972 | 0.0480877 |
| DPYSL4 | 3.47377246 | 1.55508604 | 7.75976043 | 0.00239183 |
| ANGPTL4 | 1.57127404 | 1.08864561 | 2.26786575 | 0.01579515 |
| NCAN | 3.91932541 | 1.9169112 | 8.01347064 | 0.00018169 |
| TKTL1 | 1.36032622 | 1.08199442 | 1.71025599 | 0.00842145 |
| BRS3 | 72.7687549 | 5.93395536 | 892.371339 | 0.00080135 |
| PPFIA4 | 7.40514397 | 2.34796463 | 23.3547628 | 0.0006345 |

Note: Genes with statistically prognostic value (*p*<0.05) were listed here.
