# Supplementary material for: Novel Hypoxia-Associated Gene Signature Depicts Tumor Immune Microenvironment and Predicts Prognosis of Colon Cancer Patients
Source: Front Genet. 2022 Jun 6;13:901734. doi: 10.3389/fgene.2022.901734 (PMC9208084; doi:10.3389/fgene.2022.901734)
Supplement: Supplementary file 9 [file Table4.DOCX]

Supplementary Table 4: PCR primer sequence used in the present study.

| **Gene** | **Forward Primer** | **Reverse Primer** |
| --- | --- | --- |
| β-Actin | CACCATTGGCAATGAGCGGTTC | AGGTCTTTGCGGATGTCCACGT |
| PPFIA4 | GCAGCTACTTCATGGAGTGCCT | GCCTCTTCAGACACATGATGCC |
| SERPINE1 | CTCATCAGCCACTGGAAAGGCA | GACTCGTGAAGTCAGCCTGAAAC |
| STC2 | GCATGACTTTTCTGCACAACGCT | GGCTTATGCAGCCGAACCTGTG |
