# Supplementary material for: Novel Hypoxia-Associated Gene Signature Depicts Tumor Immune Microenvironment and Predicts Prognosis of Colon Cancer Patients
Source: Front Genet. 2022 Jun 6;13:901734. doi: 10.3389/fgene.2022.901734 (PMC9208084; doi:10.3389/fgene.2022.901734)
Supplement: Supplementary file 10 [file Image1.PDF]

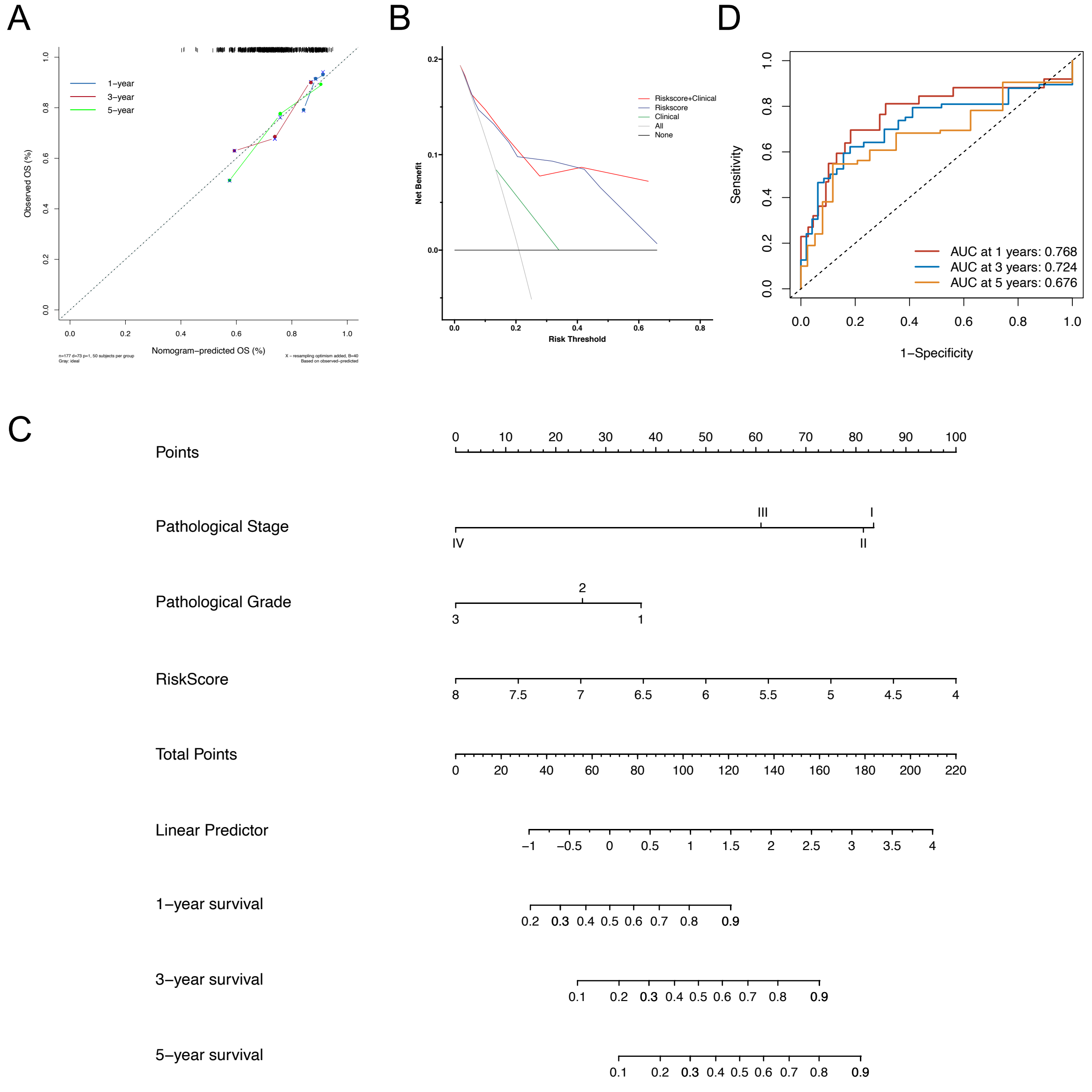
A
1−year
3−year
5−year
Observed OS (%)
Nomogram−predicted OS (%)
n=177 d=73 p=1, 50 subjects per group
Gray: ideal
X − resampling optimism added, B=40
Based on observed−predicted
B
Riskscore+Clinical
Riskscore
Clinical
All
None
Net Benefit
Risk Threshold
D
Sensitivity
1−Specificity
AUC at 1 years: 0.768
AUC at 3 years: 0.724
AUC at 5 years: 0.676
C
Points
Pathological Stage
III
I
IV
II
Pathological Grade
2
3
1
RiskScore
Total Points
Linear Predictor
1−year survival
3−year survival
5−year survival
